# Substantia nigra related gene polymorphisms associated with antipsychotic-induced acute movement disorders: a genome-wide association study and multi-ancestry validation in schizophrenia

**DOI:** 10.1186/s40779-025-00636-w

**Published:** 2025-08-19

**Authors:** Zhe Lu, Yao-Yao Sun, Zhe-Wei Kang, Guo-Rui Zhao, Yu-Yanan Zhang, Jun-Yuan Sun, Rui Yuan, Wei-Hua Yue

**Affiliations:** 1 Peking University Institute of Mental Health, NHC Key Laboratory of Mental Health (Peking University), National Clinical Research Center for Mental Disorders (Peking University Sixth Hospital), Peking University Sixth Hospital, Beijing, 100191 China; 2 PKU-IDG/McGovern Institute for Brain Research, Peking University, Beijing, 100871 China; 3 Chinese Institute for Brain Research, Beijing, 102206 China

**Keywords:** Antipsychotic, Movement disorders, Genome-wide association study (GWAS), Substantia nigra, Multi-ancestry validation

## Abstract

**Background:**

Antipsychotic-induced movement disorders (AIMDs) are prevalent side effects of antipsychotics, particularly during the acute phase of treatment. This study aimed to elucidate the genetic mechanisms underlying AIMDs using a genome-wide association study (GWAS).

**Methods:**

GWASs on AIMDs were conducted in 3 independent cohorts: a discovery cohort of 3067 patients (2016 subjects were reserved after quality control), a validation cohort of 277 patients, and a multi-ancestry validation cohort of 766 patients. Subsequent post-GWAS analyses included gene-based analyses, transcriptome-wide association studies (TWASs), and polygenic risk score (PRS) profiling.

**Results:**

Our study identified 2 loci located in *RAB44* gene (rs116249243, *P* = 5.98 × 10^–9^; rs117097482, *P* = 1.17 × 10^–8^) associated with extrapyramidal symptoms (EPSs), 1 locus (rs6826172, *P* = 5.56 × 10^–9^) related to akathisia, and 76 loci linked to involuntary movements (11 genes were mapped). Risk loci located in *CNTNAP2, LUZP2, TMEM167A*, and *RAB44* genes were successfully replicated in the validation cohort, whereas the locus located in *RAB44* was also replicated in the multi-ancestry cohort. Gene-based analyses indicated that *XRCC4* and *PAIP2B* reached significance at the genome-wide level in involuntary movements. Tissue expression analysis revealed that involuntary movement-related genes are predominantly expressed in the substantia nigra. Additionally, the TWAS suggested a causal relationship between *XRCC4* and involuntary movement. The PRSs derived from the discovery cohort significantly predicted AIMDs in the validation cohort, with area under the receiver operating characteristic curve (AUC) values from 0.60 to 0.80.

**Conclusions:**

Our findings highlight the role of substantia nigra related gene polymorphisms in AIMDs. This study provides novel insights into the pathogenesis of AIMDs and supports the potential for personalized treatment approaches for schizophrenia.

***Trial registration*:**

ChiCTR (https://www.chictr.org.cn/showproj.html?proj=8604), No. ChiCTR-TRC-10000934;

ChiCTR (https://www.chictr.org.cn/showproj.html?proj=129668), No. ChiCTR2100048320.

**Supplementary Information:**

The online version contains supplementary material available at 10.1186/s40779-025-00636-w.

## Background

Antipsychotics are essential in the management of schizophrenia. However, antipsychotics often lead to significant adverse effects (AEs), with antipsychotic-induced movement disorders (AIMDs) being the most prevalent [1]. AIMDs encompass a range of motor disturbances, including extrapyramidal symptoms (EPSs, such as tremors, acute dystonia, and Parkinsonism), akathisia, and tardive dyskinesia (TD). While it is well-established that TD usually manifests following chronic or long-term antipsychotic use, recent advancements in the fields of psychopharmacology and genetics have raised the possibility that the involuntary movements occur in the acute stage of antipsychotic treatment [2]. These motor AEs can severely impair the quality of life and secondary adherence in patients with schizophrenia, particularly during the acute phase. Although the mechanisms underlying AIMDs remain poorly understood, they are thought to arise from the complex interplay of pharmacological and genetic factors.

The symptoms associated with AIMDs are closely linked to the blockade of D_2_ receptors in the nigrostriatal pathway [2, 3]. Neuroimaging studies have indicated that the optimal occupancy rate of D_2_ receptors in the striatum ranges from 65 to 80%, and a blockade exceeding 80% may lead to AIMDs [4, 5]. Compared with first-generation antipsychotics (FGAs), second-generation antipsychotics (SGAs) can modulate excessive dopamine levels through mechanisms beyond D_2_ receptor antagonism. Consequently, patients with schizophrenia treated with SGAs experience a significantly lower risk of AIMDs than those treated with FGAs [6], which is also supported by our previous study [7]. However, a network meta-analysis, involving 136 studies and 24,911 participants, indicated that 21 of the 32 antipsychotics assessed were significantly associated with increased use of antiparkinsonian medications compared to placebo [8]. Furthermore, 20 of the 32 evaluated antipsychotics were associated with an increased risk of akathisia. Although SGAs generally present a favorable profile with a reduced risk of AIMDs compared with FGAs, careful monitoring is essential, particularly at higher doses of SGAs with high D_2_ receptor affinity [8]. Emerging evidence indicated that genetic factors significantly influence the treatment outcomes associated with antipsychotics [9]. Recent advances in genomics have facilitated the exploration of the genetic basis of drug responses and adverse drug reactions [10]. Genome-wide association studies (GWASs) are instrumental in identifying genetic variants linked to various drug-induced effects [11]. One notable GWAS examining AIMDs included 738 patients with schizophrenia in the chronic phase from the Clinical Antipsychotic Trials of Intervention Effectiveness (CATIE) in schizophrenia cohort [12]. The Simpson-Angus Scale (SAS), Barnes Akathisia Rating Scale (BARS), and Abnormal Involuntary Movement Scale (AIMS) were used to assess AIMDs and identified 3 loci that reached genome-wide significance [12]. Additionally, another GWAS investigating TD involved 1406 schizophrenia patients in remission, including samples from the CATIE cohort, and identified rs11639774 in *GSE1* as reaching genome-wide significance [13].

Research specifically targeting AIMDs is limited, with small sample sizes and a lack of studies focusing on the acute phase of antipsychotic therapy. This study aims to investigate the genetic mechanisms associated with AIMDs in a Han Chinese population with schizophrenia. We used 3 independent cohorts, including a large discovery group and 2 validation groups, to ensure robust findings. GWAS was conducted to identify genes associated with AIMDs and transcriptome-wide association study (TWAS), phenome-wide association study (PheWAS) and polygenic risk score (PRS) were further performed to provide multi-dimension evidence and reveal the underlying genetic mechanism.

## Methods

### Study design and participants

This study used 3 independent cohorts to investigate genetic associations of AIMDs. The discovery cohort consisted of 3067 patients (2016 participants were reserved after quality control) with schizophrenia in the acute phase and was derived from the Chinese Antipsychotics Pharmacogenomics Consortium (CAPOC) [14]. Participants in the CAPOC cohort were randomly assigned to 1 of 6 treatment groups: risperidone, olanzapine, quetiapine, aripiprazole, ziprasidone, or an FGA (haloperidol or perphenazine).

### Replicates

The validation cohort [paliperidone (PAL) monotherapy cohort] was a prospective multicenter clinical trial conducted from 2021 to 2023, involving 277 patients with schizophrenia in the acute phase who received PAL monotherapy for 6 weeks. Additionally, we used a multi-ancestry validation cohort from the National Institutes of Health-supported CATIE [15]. Following approval from the National Institute of Mental Health (NIMH) Repository & Genomics Resource (https://www.nimhgenetics.org/), we obtained the clinical data of 766 patients with schizophrenia in the chronic phase from CATIE cohort, which was also designed as a randomized clinical trial, with participants assigned to 5 treatment groups: perphenazine, ziprasidone, risperidone, quetiapine, and olanzapine.

### Ethics statement

All protocols involving human subjects were approved by Clinical Research Ethics Committees at each site, and written informed consent was obtained from all participants. The clinical registration numbers for the study are as follows (http://www.chictr.org.cn/index.aspx): CAPOC cohort, ChiCTR-TRC-10000934; PAL cohort, ChiCTR2100048320; CATIE cohort, Requested ID 63084551a4921.

### Data processing

**Fig. 1:**
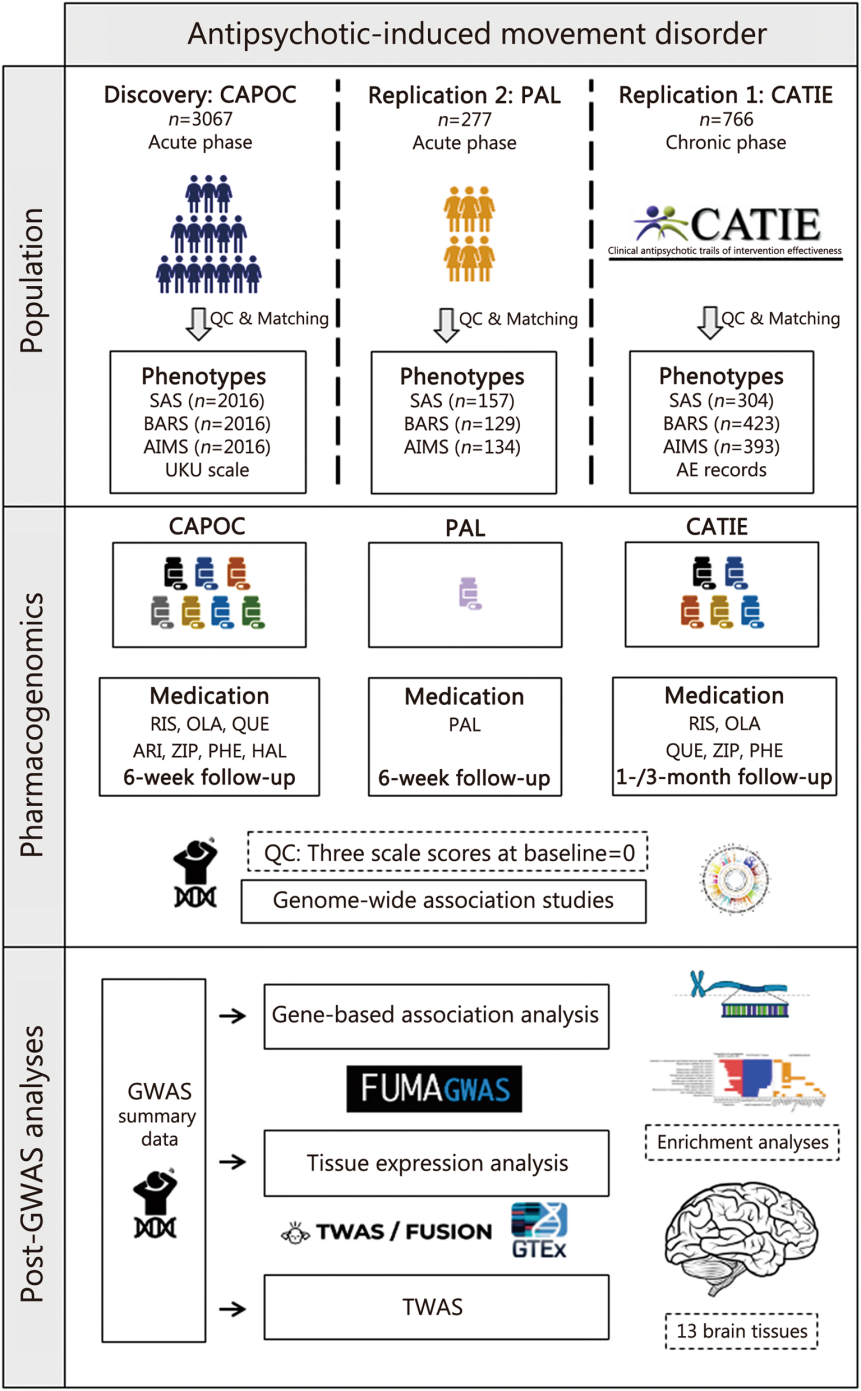
The GWASs were conducted in the discovery cohort, and the genome-level loci were validated in two independent cohorts. Several post-GWAS analyses, including enrichment analysis, gene-based association study, tissue expression, and TWAS, were also conducted. The CAPOC cohort was a discovery cohort, the validation cohort was an independent paliperidone monotherapy trial, CATIE cohort is a multi-ancestry validation cohort. CAPOC Chinese Antipsychotics Pharmacogenomics Consortium, CATIE Clinical Antipsychotic Trials of Intervention Effectiveness, PAL paliperidone, SAS Simpson-Angus Scale, BARS Barnes Akathisia Rating Scale, AIMS Abnormal Involuntary Movement Scale, RIS risperidone, OLA olanzapine, QUE quetiapine, ARI aripiprazole, ZIP ziprasidone, HAL haloperidol, PHE perphenazine, GWAS genome-wide association study, TWAS transcriptome-wide association study, QC quality control, FUMA Functional Mapping and Annotation, FUSION Functional Summary-based Imputation, GTEx Genotype-Tissue Expression

The workflow of this study is illustrated in Fig. 1. Patients in both the discovery and validation cohorts were diagnosed with schizophrenia according to the Diagnostic and Statistical Manual of Mental Disorders, Fourth Edition, and were of Han Chinese ancestry. Outcomes were assessed at baseline and the 2nd, 4th, and 6th weeks in both cohorts. To validate the findings of the CAPOC cohort, we extracted data from the CATIE cohort, focusing on measurements taken at baseline and the 1st and 3rd weeks. Detailed enrollment procedures, randomization, masking, clinical protocols, and data collection methods are provided in the Additional file 1.

The SAS, BARS, and AIMS were used to assess AIMDs. The total scores of SAS were used to assess EPSs, the total scores of BARS were used to assess the akathisia, and the total scores of AIMS were used to assess the involuntary movements (only the total scores of the first 7 items were calculated). Change scores from baseline to week 6 for these 3 scales were considered as phenotypes. Patients with baseline scores more than 0 on these scales were excluded. Additionally, the Utvalg for Kliniske Undersogelser (UKU) side effect scale in the CAPOC cohort and AE records in the CATIE cohort were employed to validate the significant findings.

Samples from the CAPOC cohort were genotyped using Illumina Human Omni ZhongHua-8 BeadChips (Illumina, San Diego, CA, USA), specifically designed for Chinese populations. PAL cohort samples were genotyped using the Infinium Asian Screening Array (Illumina, San Diego, CA, USA), tailored for East Asian populations. Genotype imputation was performed to identify missing single-nucleotide polymorphisms (SNPs). Samples were excluded if the genotype call rate < 98%, gender discordance was detected, they were first- or second-degree relatives, or identified as genetic outliers. SNPs were excluded based on a minor allele frequency < 0.01, a genotype call rate < 98%, or *P-*values for Hardy–Weinberg equilibrium < 1 × 10^–5^. Genotype imputation utilized a pre-phasing stepwise approach implemented in IMPUTE2 and SHAPEIT (version 2). For the CATIE cohort, genotyping was conducted using the Affymetrix 500 K “A” chip (Santa Clara, CA, USA) and the Perlegen custom 164 K chip. Additional details on genotyping, genetic quality control, and imputation methods for the CAPOC and validation cohorts are provided in the Additional file 2.

### Statistical analysis

The GWAS of AIMDs in the CAPOC cohort was conducted using the PLINK software (v1.90, http://pngu.mgh.harvard.edu/purcell/plink/) [16]. Linear regression analyses were employed to evaluate the associations between alleles and AIMDs, controlling for covariates including gender, age, research center, type and dosage of antipsychotics (converted to chlorpromazine equivalent doses), duration of schizophrenia at baseline, and the first 5 principal components to account for population structure. We hypothesized that the effects of different antipsychotics on movement disorders would converge on general pathways and assessed allele associations across the entire sample. A genome-wide significance threshold of *P* < 5 × 10^–8^ was established, with *P* < 1 × 10^–5^ indicating findings of interest.

The same procedure was used for the PAL cohort. In the CATIE cohort, GWASs were conducted separately for the 3 populations, followed by meta-analyses using the METAL tool. A *P-*value < 0.05 in the PAL and CATIE cohorts was considered indicative of satisfactory replication. To further confirm the association between genome-wide loci (*P* < 5 × 10^–8^) and AIMDs, we conducted logistic regression analyses in the CAPOC cohort to examine the associations between these risk loci and items on the UKU, including dystonia, rigidity, hypokinesia, hyperkinesia, tremor, and akathisia. Additionally, we assessed the association between the risk loci and AE records, specifically focusing on akathisia and akinesia.

Genome-wide significant loci were initially mapped to genes, and the expression quantitative trait loci (eQTL) effects of the identified SNPs and the expression patterns of relevant genes in human tissues were explored, with a primary focus on brain tissues, using the Genotype-Tissue Expression database (https://www.gtexportal.org/). Subsequently, all genes with *P* < 1 × 10^–5^ underwent Gene Ontology enrichment analysis to identify overrepresented biological processes, along with tissue specificity analysis conducted via the Functional Mapping and Annotation (FUMA) online tool (https://fuma.ctglab.nl/) to assess expression levels across different tissues [17]. For each phenotype, gene-based and tissue expression analyses were performed using Multi-marker Analysis of GenoMic Annotation through FUMA to enhance GWAS power. Additionally, TWASs were executed using the FUSION tool (http://gusevlab.org/projects/fusion/), inferring causal relationships between genes and phenotypes (a total of 60,628 genes from 13 brain tissues were included, with a *P* threshold level = 0.05/60628 = 8.25 × 10^–7^). Finally, all risk loci were subjected to the PheWASs [18] using the GWAS-Atlas website (https://atlas.ctglab.nl./) to investigate associations between risk loci and a broad spectrum of phenotypes, particularly focusing on Parkinson’s disease [19].

PRSs were calculated using the PRSice-2 tool [20]. Reference data were derived from the summary statistics of 3 CAPOC phenotypes, and target data were obtained from the PAL cohort. We established 5 thresholds (1 × 10^–5^, 1 × 10^–3^, 1 × 10^–2^, 5 × 10^–2^, and all SNPs) to assess the associations between PRSs and change scores across 3 scales.

To develop a prediction model for AIMDs, we employed logistic regression analyses and tested its discriminatory ability in the PAL cohort. Cases were defined as samples with change scores > 0 on the 3 scales at the 2nd, 4th, and 6th weeks. Samples with total baseline scores > 0 were excluded from the analyses. All PRS levels were included in the logistic regression analysis using the forward selection method. The discrimination performance of the model was assessed using the area under the receiver operating characteristic curve (AUC). The sensitivity, specificity, accuracy, positive predictive value, and negative predictive value were also calculated.

## Results

### Demographic and clinical characteristics of samples

There were no significant differences in age, gender, onset age, proportion of first episode, or disease duration among the different therapy groups in the CAPOC cohort. Following data cleaning, the GWAS in the CAPOC cohort comprised 2016 participants with complete genetic and clinical data. In the PAL cohort, after data cleaning, 157 patients had both genetic and SAS data, 129 had genetic and BARS data, and 134 had both genetic and AIMS data. Similarly, in the CATIE cohort, data cleaning yielded 304 patients with genetic and SAS data, 423 with genetic and BARS data, and 393 with genetic and AIMS data. Detailed demographic data are presented in Additional file 3: Tables S1, S2, and Additional file 1: Fig. S1 illustrates the data cleaning pipeline.

### Identification of novel loci associated with AIMDs in Chinese Han populations

**Fig. 2:**
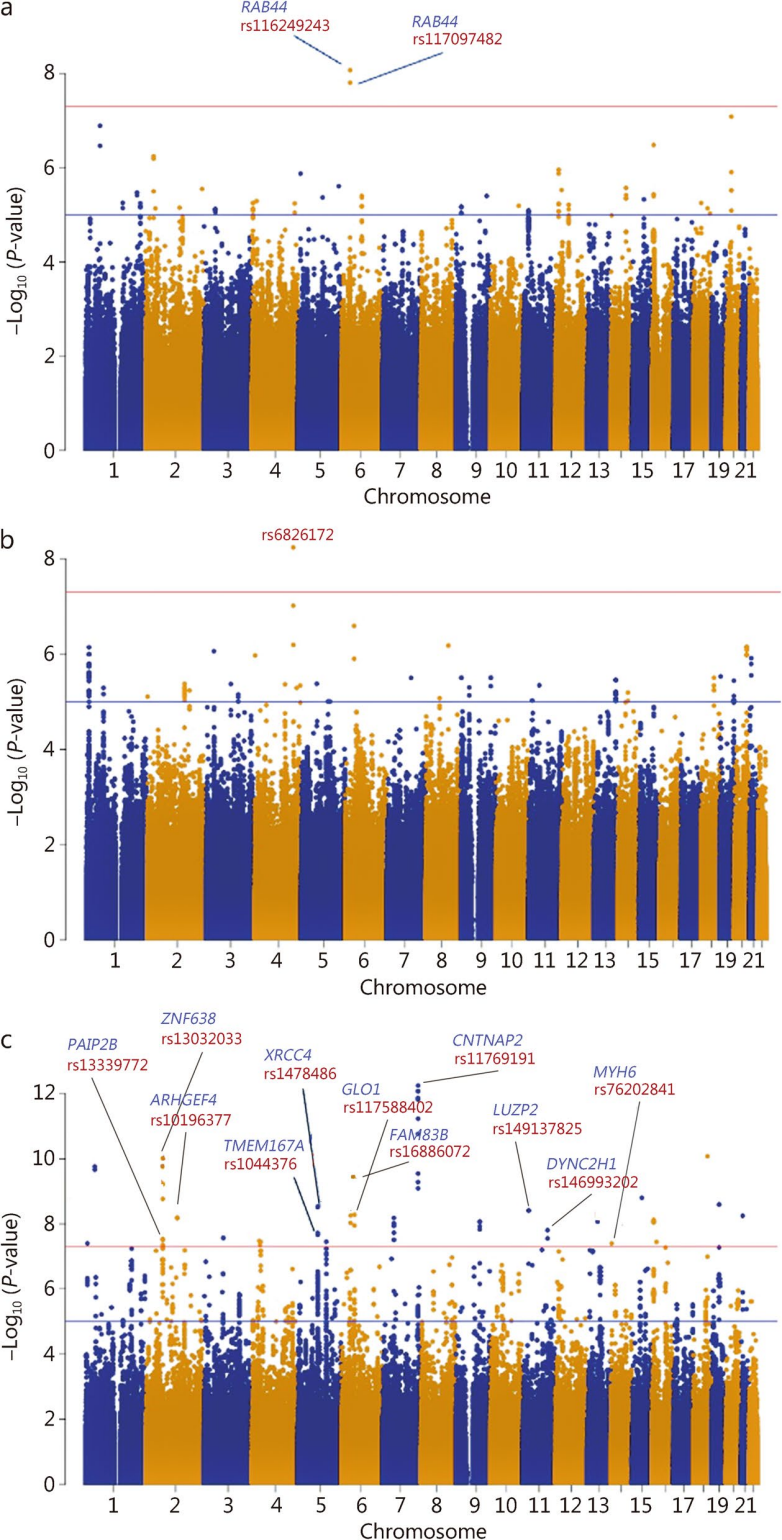
Manhattan plots of 3 movement disorder phenotypes in the discovery cohort. **a** Manhattan plot of the GWAS results of SAS. **b** Manhattan plot of the GWAS results of BARS. **c** Manhattan plot of the GWAS results of AIMS. SAS Simpson-Angus Scale, BARS Barnes Akathisia Rating Scale, AIMS Abnormal Involuntary Movement Scale, GWAS genome-wide association study

In the discovery cohort, 2016 samples with 4,609,700 genotyped SNPs were included in the GWAS. For the SAS, we identified 2 loci in *RAB44* at a genome-wide significance level (rs116249243, *P* = 5.98 × 10^–9^; rs117097482, *P* = 1.17 × 10^–8^) (Fig. 2a; Additional file 3: Table S3). For the BARS, 1 locus reached genome-wide significance (rs6826172, *P* = 5.56 × 10^–9^) (Fig. 2b; Additional file 3: Table S3). Notably, for the AIMS, we identified 76 loci at the genome-wide significance level, mapping to 11 genes, including *CNTNAP2, ZNF638, LUZP2, FAM83B, GLO1, ARHGEF4, DYNC2H1, XRCC4, TMEM167A, MYH6,* and *PAIP2B* (Fig. 2c; Additional file 3: Table S3). All of these loci were not at a genome-wide significant level in the summary data of schizophrenia in the East Asian population, which indicated that the findings are specific to medication-related side effects.

### Neurodevelopmental pathways enriched in AIMDs

**Fig. 3:**
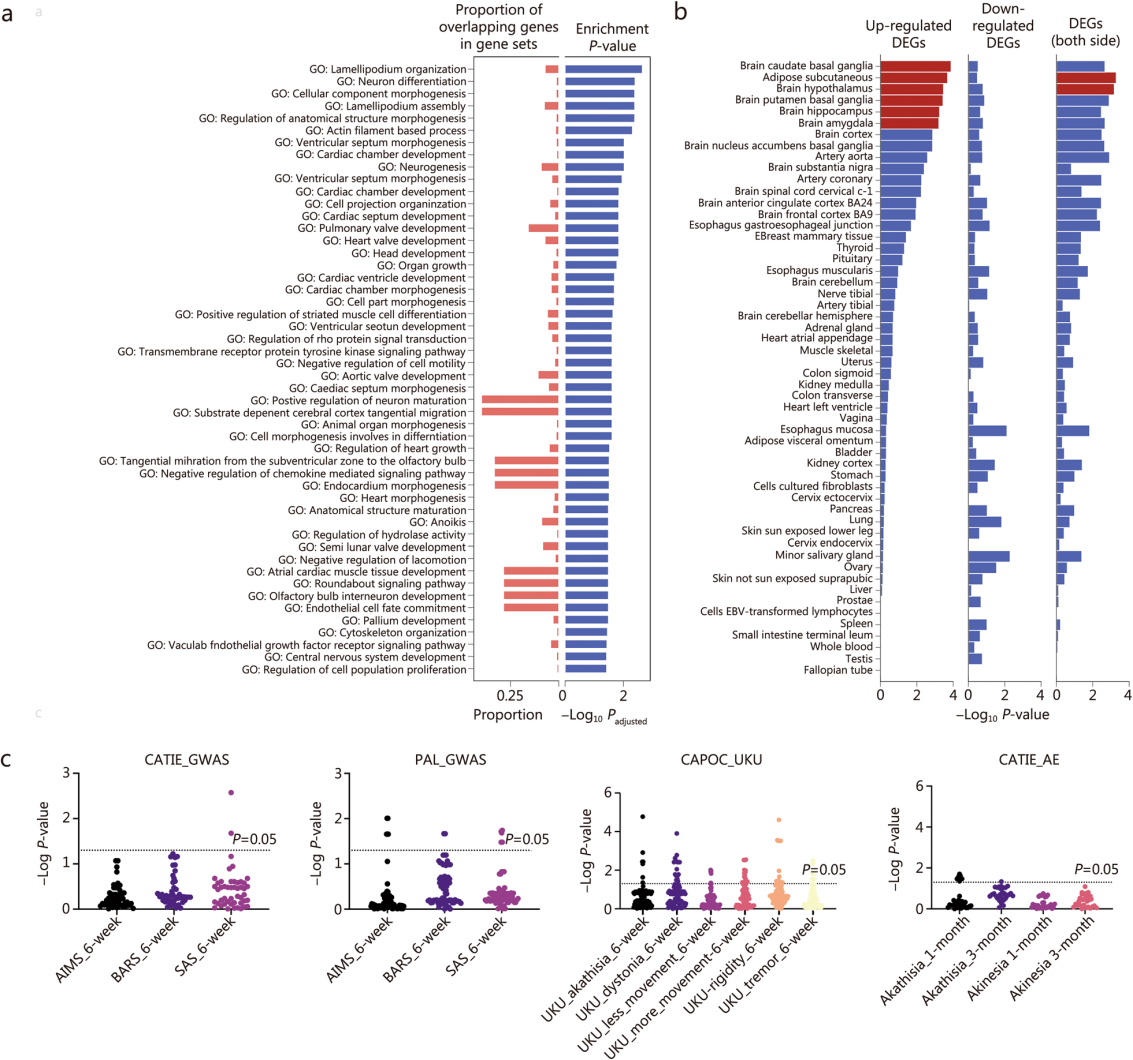
Enrichment and tissue specificity analyses on genome-level loci in the discovery cohort, and GWAS results validation in 2 independent cohorts. **a** Enrichment result of loci with a *P-*value < 1 × 10^–5^. **b** Tissue specificity results of loci with *P-*value < 1 × 10^–5^, red bars indicate tissue specificity results that reached statistical significance. **c** Validation analyses in 2 independent cohorts and 2 other phenotypes. CAPOC Chinese Antipsychotics Pharmacogenomics Consortium, CATIE Clinical Antipsychotic Trials of Intervention Effectiveness, PAL paliperidone, SAS Simpson-Angus Scale, BARS Barnes Akathisia Rating Scale, AIMS Abnormal Involuntary Movement Scale, UKU Utvalg for Kliniske Undersogelser, AE adverse effect, GWAS genome-wide association study, DEGs differentially expressed genes

SNPs with a *P-*value < 1 × 10^–5^ were defined as suggestive SNPs, and the related genes were defined as suggestive genes. We first checked the overlap of these suggestive results among the 3 phenotypes. We found that 1 overlapping gene (*RAB44*) overlapped between the SAS and BARS, 3 overlapping genes (*CTNND2, STK32B, MYH6*) overlapped between the SAS and AIMS, and 22 overlapping SNPs overlapped between the results of the BARS and AIMS (Additional file 1: Figs. S2, S3; Additional file 3: Tables S4–S6). Subsequently, we conducted enrichment analysis using 101 suggestive genes of the discovery cohort, revealing that significant pathways involved in neurodevelopment (neuron differentiation, *P*_adjusted_ = 4.03 × 10^–3^; neuron development, *P*_adjusted_ = 9.48 × 10^–3^; neurogenesis, *P*_adjusted_ = 9.48 × 10^–3^; forebrain development, *P*_adjusted_ = 1.45 × 10^–2^; neuron maturation, *P*_adjusted_ = 2.47 × 10^–2^) were enriched (Fig. 3a). Furthermore, these suggestive genes were specifically expressed in the brain tissue (Fig. 3b).

### Independent sample validation confirms risk loci for AIMDs

In the PAL cohort (the validation cohort), 3,496,532 genotyped SNPs were included. Five risk loci associated with AIMS in the discovery cohort were successfully replicated in the SAS phenotype (rs73460109 located in *CNTNAP2*, *P_PAL_SAS* = 1.82 × 10^–2^; rs76413600 located in *CNTNAP2*, *P_PAL_SAS* = 2.07 × 10^–2^; rs79657351 located in *CNTNAP2*, *P_PAL_SAS* = 1.94 × 10^–2^; rs144492884 and rs149137825 located in *LUZP2*, both *P_PAL_SAS* = 3.32 × 10^–2^). Additionally, 2 risk loci for the AIMS in the discovery cohort were replicated in the BARS phenotype (rs144492884 and rs149137825 located in *LUZP2*, both *P_PAL_BARS* = 2.15 × 10^–2^), and 5 risk loci for the AIMS in the discovery cohort were replicated in the AIMS phenotype (rs1827697 and rs1875992, *P_PAL_AIMS* = 9.83 × 10^–3^; rs1044376, rs1044374, and rs1044363 located in *TMEM167A*, all *P_PAL_AIMS* = 2.20 × 10^–2^).

In the CATIE cohort (the multi-ancestry cohort), which included 5,159,546 genotyped SNPs, 1 risk locus for the AIMS in the discovery cohort (rs117082492, *P_CATIE_SAS* = 2.67 × 10^–3^) and 1 risk locus for the SAS in the discovery cohort (rs116249243 located in *RAB44*, *P_CATIE_SAS* = 2.11 × 10^–2^) were successfully replicated in the SAS phenotype (Fig. 3c; Additional file 3: Table S3).

To validate the significant association between risk loci and AIMDs, we assessed the correlation between risk loci and related items of the UKU in the CAPOC cohort and between risk loci and related items of AE records in the CATIE cohort. Our analyses indicated that the associations between risk loci and clinically observed movement disorders were widely significant in the CPAOC cohort, and the most pronounced correlation was observed between rs6826172 and akathisia (*P* = 1.68 × 10^–5^; rs6826172 was the top SNP in GWAS of BARS, *P* = 5.56 × 10^–9^). Similarly, an association between these loci and akathisia was also confirmed in the CATIE cohort (Fig. 3c; Additional file 3: Tables S7, S8).

### Multi-omics convergence points to substantia nigra in AIMDs

**Fig. 4:**
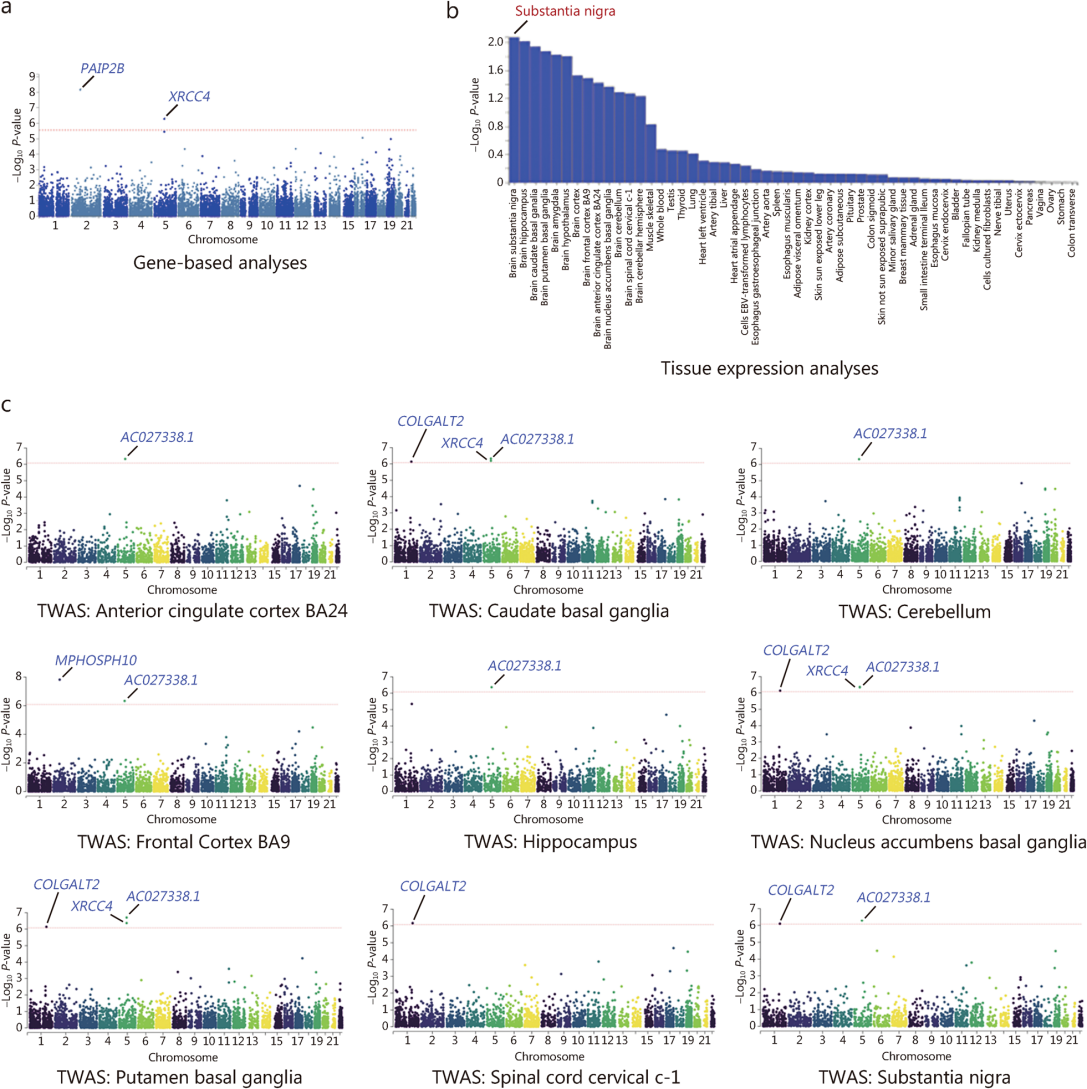
Post-GWAS analyses on the GWAS results of AIMS.** a** Gene-based analysis. **b** Tissue expression analysis. **c** TWAS based on brain tissues from the GTEx database. AIMS Abnormal Involuntary Movement Scale, TWAS transcriptome-wide association study, GWAS genome-wide association study

To augment the significance of the GWAS, several secondary analyses were conducted, with significant findings emerging exclusively from AIMS results. The gene-based analysis, providing evidence from the gene-level, revealed that both the *PAIP2B* and *XRCC4* genes attained genome-wide significance (involved in 18,053 protein-coding genes, *P*_adjusted_ = 2.77 × 10^–6^) (Fig. 4a). Additionally, multi-marker analysis of GenoMic annotation tissue expression analysis demonstrated that genes associated with involuntary movements were specifically expressed in the substantia nigra, a key region implicated in the pathogenesis of AIMDs (Fig. 4b).

The TWAS, with default settings to further explore the expression pattern of genetic variants for AIMD results, showed that *XRCC4* exhibited causal relationships (caudate basal ganglia, *P_TWAS* = 6.36 × 10^–7^ nucleus accumbens basal ganglia, *P_TWAS* = 4.51 × 10^–7^; putamen basal ganglia, *P_TWAS* = 1.97 × 10^–7^) with involuntary movements (Fig. 4c; Additional file 3: Table S9).

Finally, we conducted PheWASs to investigate the associations between risk loci and a broad spectrum of phenotypes, particularly focusing on Parkinson’s disease given the clinical similarities between Parkinson’s disease and AIMDs (Additional file 1: Fig. S4). PheWASs indicated that *ZNF638* (Parkinson’s disease of sibling pairs, *P* = 6.12 × 10^–3^)*, LUZP2* (Parkinson’s disease of sibling pairs, *P* = 3.26 × 10^–2^)*, FAM83B* (Parkinson’s disease, *P* = 8.99 × 10^–3^)*, GLO1* (familial Parkinson’s disease, *P* = 4.20 × 10^–2^)*,* and *MYH6* (Parkinson’s disease, *P* = 1.35 × 10^–2^) were associated with Parkinson’s disease (Additional file 1: Fig. S4; Additional file 3: Table S10).

### Integration of PRS with clinical covariates enhances precision in AIMD’s risk

**Fig. 5:**
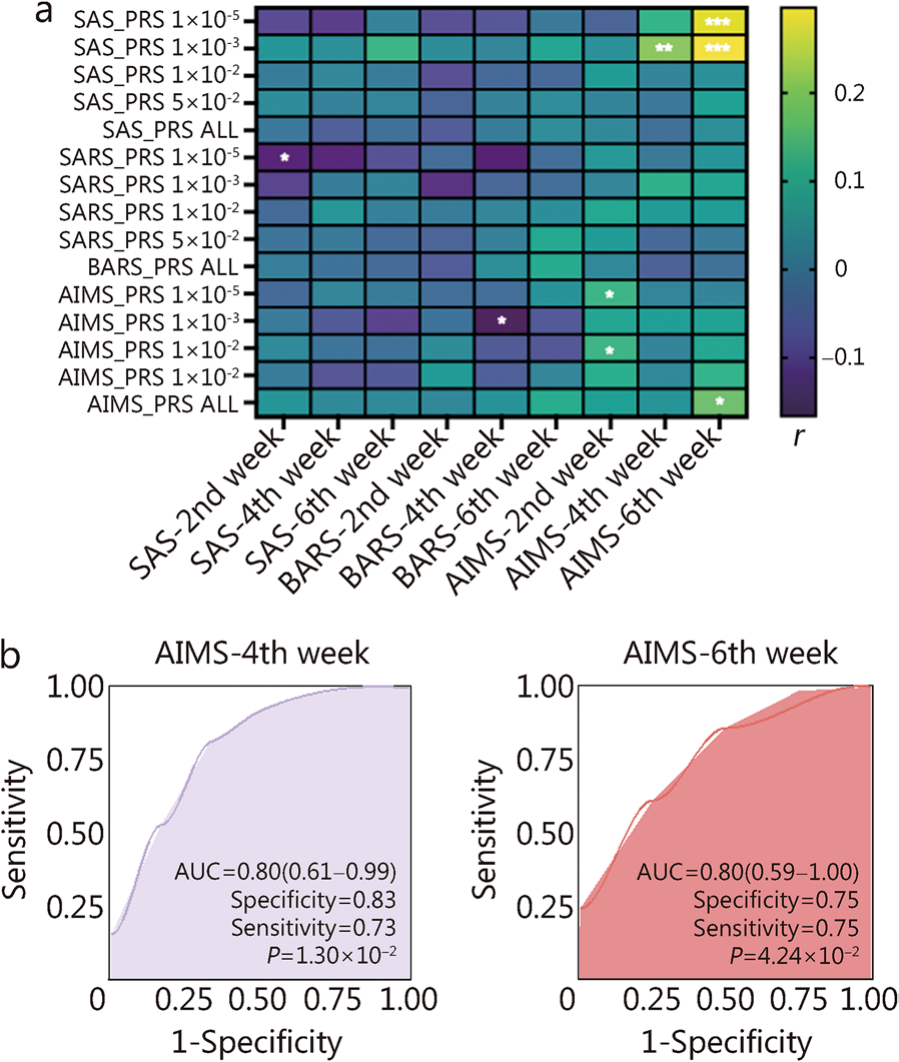
Polygenetic risk scores (PRSs) for prediction of antipsychotic-induced movement disorders in the PAL cohort.** a** Associations between the PRSs based on the CAPOC cohort and 3 scores in the PAL cohort. **b** Area under the curve of PRS predictors for AIMS scale in PAL cohort. ^*^P < 0.05, ^**^P < 0.01, ^***^P < 0.001. CAPOC Chinese Antipsychotics Pharmacogenomics Consortium, SAS Simpson-Angus Scale, BARS Barnes Akathisia Rating Scale, AIMS Abnormal Involuntary Movement Scale, AUC area under the receiver operating characteristic curve

The correlations between the PRSs of AIMDs based on the discovery cohort and the changing scores of the 3 scales after antipsychotic treatment were detected in the PAL cohort. The PRS__SAS_ at 1 × 10^–5^ and 1 × 10^–3^ levels, and PRS__AIMS,_ including all SNPs, were significantly associated with the total scores of AIMS at the 6th week. Additionally, the PRS__SAS_ at 1 × 10^–3^ level was associated with the total scores of AIMS at 4th week, the PRS__AIMS_ at 1 × 10^–5^ and 1 × 10^–2^ level were significantly associated with the total scores of AIMS at 2nd week. Moreover, PRS__AIMS_ at 1 × 10^–3^ level was associated with the total scores of BARS at 4th week, and the PRS__BARS_ at 1 × 10^–5^ level was significantly demonstrated a significant correlation with the total scores of SAS at the 2nd week (Fig. 5a).

The PRSs exhibited good discrimination for AIMDs, particularly the predictive capability of PRS__SAS_1E-03_ concerning involuntary movement [at the 4th week, AUC = 0.80, 95% confidence interval (CI) = 0.61 − 0.99, specificity = 0.83, sensitivity = 0.73; at the 6th week, AUC = 0.80, 95% CI = 0.59 − 1.00, specificity = 0.75, sensitivity = 0.75] (Fig. 5b**);** the predictive performance of PRSs for BARS and SAS were described in Additional file 1: Fig. S5; Additional file 3: Table S11. Then we incorporated age, sex, and PAL dose as clinical covariates into our predictive model for AIMDs. This adjustment improved the model’s predictive performance (the predictive capability of PRS__SAS_1E-03_ concerning involuntary movement at the 4th week, adjusted AUC = 0.83; at the 6th week, adjusted AUC = 0.96; Additional file 3: Table S11).

We finally conducted a sensitivity analysis to address the potential impact of the exclusion of participants with any baseline AIMD score > 0. The results showed that the difference was significant, which may reflect the low risk of AIMDs associated with SGAs. Notably, the significant difference of change scores of AIMS was not observed in the haloperidol subgroup (Additional file 3: Table S12).

## Discussion

To the best of our knowledge, this study is the largest-scale GWAS of AIMDs, GWAS of AIMDs in the Chinese Han population, and GWAS of AIMDs in the acute phase. Our study identified 79 novel loci associated with AIMDs. Importantly, the implicated risk genes are specifically expressed in the substantia nigra, a critical region involved in the pathogenesis of AIMDs. Our findings underscore the significant role of neurodevelopmental pathways and substantia nigra in the underlying mechanisms of AIMDs. Additionally, the PRSs derived from this GWAS demonstrated promising predictive power for AIMDs, offering potential utility for early identification and intervention.

*RAB44*, which is a member of the RAS oncogene family and is involved in Guanosine Triphosphate binding, GTPase activity, and calcium ion binding [21], and plays a role in innate immunity [22], was found to be associated with EPSs. A recent scoping review examined skeletal muscle abnormalities and motor dysfunction in patients with schizophrenia, which may be correlated with elevated creatine kinase levels [23]. Antipsychotics may further exacerbate these issues [23], potentially because of prolonged periods of skeletal muscle abnormalities resulting from acute dystonia. An animal study suggested that *Rab44* could affect skeletal muscle recovery from damage by regulating mechanistic target of rapamycin complex 1 signaling and the transport of fusogenic regulators [24], offering a potential link between *RAB44* and EPSs. The polymorphism rs6826172 was associated with akathisia. Although rs6826172 is not located within a gene, its eQTL effects reveal that it affects the expression of the *GLRB* gene in brain tissue [25, 26]. The protein encoded by *GLRB* is a crucial component of the isoforms of the ligand-gated chloride channel, playing a vital role in downregulating neuronal excitability and contributing to the neurotransmitter receptors and postsynaptic signaling pathways involved in the generation of inhibitory postsynaptic currents [27]. Additionally, mice with *Glrb* gene mutations serve as classic models of spasms [28]. Gene set analysis results indicated that the genes associated with akathisia were enriched in the pathway of voluntary skeletal muscle movements. Given the clinical comorbidity of EPSs and akathisia, these findings were consistent with our expectations. Moreover, the GWAS results indicated that rs116249243 was significant in both SAS (*P* = 5.98 × 10^–9^) and BARS (*P* = 2.61 × 10^–7^). This indicated that a shared mechanism exists between the 2 phenotypes. Patients often present with multiple concurrent AIMDs in clinical practice, and studying this subgroup is indeed crucial for a more comprehensive understanding of antipsychotic-related risks.

In this study, 76 SNPs associated with involuntary movement were identified. The most significant locus, rs11769191, is located within the *CNTNAP2* gene, which also exhibits an eQTL effect on the *CNTNAP2* in brain tissues. *CNTNAP2* encodes a neuronal surface protein that is implicated in cell adhesion and nervous system development. It regulates the interactions between neurons and glial cells and plays a crucial role in the localization of potassium channels in axonal differentiation during nervous system development [29]. *CNTNAP2* is associated with various neurodevelopmental disorders, including schizophrenia, epilepsy, autism, attention-deficit hyperactivity disorder [30], and intellectual disabilities [31, 32]. *Cntnap2*^−/−^ mice display the core behaviors of autism, hyperactivity, and seizure. Additionally, *CNTNAP2* is linked to severe depressive, psychotic, and agitation symptoms, and it can increase the excitability of hippocampal neurons, promoting seizure-like activity [33]. *Cntnap2* mutant mice displayed characteristic delays in myelination and stereotypical motor behaviors [34, 35]. The *CNTNAP2* gene is also associated with anti-contactin-associated protein receptor 2 encephalitis, a type of autoimmune encephalitis, in which some patients exhibit Parkinsonian symptoms and ataxia [36–38]. Both *LUZP2* and *GLO1* are highly expressed in the substantia nigra, a region implicated in the pathogenesis of AIMDs. Several studies have suggested that *GLO1* inhibitors may serve as promising therapeutic agents for mental disorders [39–41]. Moreover, the *GLO1* gene is associated with restless legs syndrome, which is relatively common in patients receiving antipsychotics [42]. *GLO1* is also a drug target of baicalein and indomethacin, which are emerging as preclinical candidates for Parkinson’s disease management [43, 44]. The *MYH6* gene, which encodes myosin heavy chain 6, is associated with muscle contraction and is highly expressed in skeletal muscle tissues. Notably, *LUZP2, CLO1,* and *MYH6* genes were also associated with Parkinson’s disease in PheWASs.

In our GWASs and gene-based analyses, *PAIP2B* and *XRCC4* were both significant at the genome-wide significance level. Notably, *XRCC4* was significant in TWASs, underscoring its relevance in this context. *PAIP2B* encodes poly(A) binding protein interacting protein 2B and is highly expressed in the substantia nigra, suggesting its role in neuronal function. *XRCC4* encodes X-ray repair cross-complementing 4, a crucial scaffold protein that facilitates the recruitment of other proteins to DNA double-strand breaks [45]. DNA double-strand breaks are common in rapidly dividing cells, such as proliferating progenitors during central nervous system development, and *Xrcc4* deficiency in mice results in heightened sensitivity to radiation, growth defects, and the loss of a substantial proportion of post-mitotic neurons [46, 47]. Additionally, SNPs in *XRCC4* are also related to the white matter microstructure [48].

Our findings suggested that PRSs based on AIMDs could predict the occurrence of movement disorders in the acute stage, as demonstrated in an independent cohort. However, its predictive performance was not sufficiently robust, indicating the need for additional pharmacogenetic studies to better capture the effect of medications on outcomes. This evidence indicated that these risk genes are promising candidate targets for the treatment of AIMDs. The modest AUC values underscore that AIMDs are multifactorial, with additional contributions from demographic, pharmacological, and environmental factors. However, here we intentionally restricted our scope to genetic predisposition in order to clarify its independent role. We aimed to isolate and quantify the predictive contribution of genetic factors (via PRS) to AIMD risk, rather than to build a comprehensive clinical prediction model incorporating non-genetic variables.

We identified several SNPs related to the AIMS while we found only a few SNPs associated with the SAS and BARS, which might explain the multifactorial nature of tardive syndromes (or involuntary movements) and difficulties of their management (or treatment refractoriness). Nonetheless, further investigations, particularly clinical trials, are essential to determine whether these genes can serve as viable therapeutic targets for AIMDs. The pathophysiology underlying AIMDs remains poorly understood, but it is potentially linked to the blockade of dopamine D_2_ receptors in the nigrostriatal pathway. Many of the identified risk genes are specifically expressed in the substantia nigra, suggesting their contribution to the pathogenesis of AIMDs. Identification of novel risk genes in this study provides a critical opportunity to advance our understanding of the pathophysiology of AIMD. Further mechanistic investigations and functional characterizations of these risk genes will be instrumental in elucidating the neurobiology of AIMDs and may reveal new therapeutic avenues.

This study had several limitations. First of all, although we included 8 antipsychotics in our analysis, we did not consider other antipsychotics that are known to contribute significantly to AIMDs, such as amisulpride, which is widely used in clinical practice. Future studies should aim to analyze the genetic effects of a broader range of antipsychotics. Additionally, most participants in this study were not antipsychotic-naive, and the allowance for antiparkinsonian medication represented a significant confounder in our results. Therefore, pharmacogenomic studies involving first-episode antipsychotic-naive patients with schizophrenia are warranted. Moreover, PAL is an active metabolite of risperidone, which means that PAL shares a similar pharmacological profile with risperidone, which was one of the medications used in the discovery cohort. By using PAL in the replication cohort, the study can maintain a degree of continuity with the discovery cohort. However, this approach introduces a limitation to the study’s ability to fully replicate the original results. In addition, the severity of AIMDs and the dynamic clinical process were overlooked. Excluding patients with any baseline AIMDs (score > 0) may have omitted a clinically relevant subgroup; it will be addressed in future studies by analyzing the trajectories of AIMDs, including dose adjustments and severity escalation. Although this was the largest GWAS conducted on AIMDs to date, the sample size remained limited, highlighting the urgent need for additional pharmacogenomic studies using larger cohorts.

## Conclusions

In summary, we identified novel risk loci, potential causal variants, and genes associated with AIMDs. These findings enhance our understanding of the genetic architecture underlying AIMDs and highlight promising therapeutic targets for future studies.

## Supplementary Information

**Additional file 1.** Methods. **Fig. S1** Pipeline of the GWAS. **Fig. S2** Venny plot of suggestive SNPs. **Fig. S3** Venny plot of suggestive genes. **Fig. S4** PheWAS results. **Fig. S5** Area under the curve of PRS predictors for SAS and BARS scales in the PAL cohort.

**Additional file 2.** Protocol of the Chinese Antipsychotics Pharmacogenomics Consortium (CAPOC) cohort.

**Additional file 3.** **Table S1** Demographic data of CAPOC. **Table S2** Demographic data of CATIE and PAL. **Table S3** Results of GWAS and validation. **Table S4** Results of GWAS on SAS (*P* < 1 × 10^-5^). **Table S5** Results of GWAS on BARS (*P* < 1 × 10^-5^). **Table S6** Results of GWAS on AIMS (*P* < 1 × 10^-5^). **Table S7** Results of validation on UKU. **Table S8** Results of validation on AE records. **Table S9** Results of TWAS. **Table S10** Results of PheWAS. **Table S11** Results of the prediction model. **Table S12** Differences of the AIMS risk between the two subgroups according to the baseline scale scores.

## Data Availability

The data that support the findings of this study are available on request from the corresponding author upon reasonable request.

## Abbreviations

AEs: Adverse effects
AIMDs: Antipsychotic-induced movement disorders
AIMS: Abnormal involuntary movement scale
AUC: Area under the receiver operating characteristic curve
BARS: Barnes akathisia rating scale
CAPOC: Chinese antipsychotics pharmacogenomics consortium
CATIE: Clinical antipsychotic trials of intervention effectiveness
CI: Confidence interval
EPSs: Extrapyramidal symptoms
eQTL: Expression quantitative trait loci
FGAs: First-generation antipsychotics
FUMA: Functional mapping and annotation
GWAS: Genome-wide association study
PAL: Paliperidone
PheWASs: Phenome-wide association studies
PRS: Polygenic risk scores
SAS: Simpson-angus scale
SNPs: Single-nucleotide polymorphisms
SGAs: Second-generation antipsychotics
TD: Tardive dyskinesia
TWAS: Transcriptome-wide association studies
UKU: Utvalg for Kliniske Undersogelser
