# Supplementary material for: Substantia nigra related gene polymorphisms associated with antipsychotic-induced acute movement disorders: a genome-wide association study and multi-ancestry validation in schizophrenia
Source: Mil Med Res. 2025 Aug 19;12:50. doi: 10.1186/s40779-025-00636-w (PMC12362978; doi:10.1186/s40779-025-00636-w)
Supplement: Supplementary file 1 — Additional file 1. Methods. Fig. S1 Pipeline of the GWAS. Fig. S2 Venny plot of suggestive SNPs. Fig. S3 Venny plot of suggestive genes. Fig. S4 PheWAS results. Fig. S5 Area under the curve of PRS predictors for SAS and BARS scales in the PAL cohort. [file 40779_2025_636_MOESM1_ESM.pdf]

Methods

Discovery cohort

Subject enrollment

This study was conducted in accordance with the Declaration of Helsinki. The protocol was approved by the institutional ethics review boards at each site, and written informed consent was obtained.

Inclusion criteria

The following conditions need to be met. (1) Diagnosed with schizophrenia based on the Structured Clinical Interview of the Diagnostic and Statistical Manual of Mental Disorders, fourth edition (DSM-IV); (2) Aged from 18 to 45 years; (3) Han Chinese ancestry; (4) Total scores of the Positive and Negative Syndrome Scale (PANSS) > 60, and 3 positive items scored > 4 at least; (5) Physically healthy with all laboratory parameters within normal limits; (6) Could be treated with oral medication; and (7) Provide informed consent.

Exclusion criteria

Patients were excluded from the study if they met the following criteria: (1) Diagnosed with other mental disorders except for schizophrenia, met the criteria of DSM-IV; (2) With unstable physical diseases, malignant syndrome or acute dystonia, well-documented histories of epilepsy and hyperpyretic convulsion; (3) Required long-acting injectable antipsychotics; (4) Regularly took with clozapine during the past month; (5) Treated with electroconvulsive therapy during the last month; (6) Had previously attempted suicide, or had experienced the symptoms of severe excitement and agitation; (7) Abnormal liver or renal function; (8) Without a legal guardian; (9) Had QT interval correction (QTc) prolongation, a history of congenital QTc prolongation, or myocardial infarction within the past

6 months; (10) Pregnant or breastfeeding; and (11) Had a contraindication to any of the drugs to which they could be assigned. Eligible patients were randomly assigned to 7 groups and received 6-week antipsychotic treatment.

Randomization and masking

Eligible patients were randomly assigned (1:1:1:1:1:1) to 6 groups [aripiprazole, olanzapine, quetiapine, risperidone, ziprasidone, or one of the first-generation antipsychotics (haloperidol or perphenazine)]. Those randomly assigned to the first-generation antipsychotics group were subsequently randomly assigned (1:1) to haloperidol or perphenazine. Group assignment was established with a Microsoft Excel randomization generator without any stratification factors. The random allocation sequence was generated by a trained research assistant who had no further role in the trial and was concealed until after baseline assessments. The researchers doing both the baseline and the follow-up assessments were masked to the group assignments of each participant. Patients and psychiatrists were unmasked to the assigned antipsychotics.

Clinical procedure

All patients were given a screening questionnaire, which recorded demographic and clinical information. We did baseline assessments to ensure that participants met the inclusion criteria. Patients who were already taking antipsychotic medications were obliged to switch to their newly assigned drug within 1 week of randomization. Within 2 weeks of randomization, psychiatrists from the study adjusted the dosages based on treatment effectiveness, in keeping with the study protocol (olanzapine doses could range from 5 to 20 mg per day, risperidone from 2 to 6 mg per day, quetiapine from 400 to 750 mg per day, aripiprazole from 10 to 30 mg per day, ziprasidone from 80 to 160 mg per day, haloperidol from 6 to 20 mg per day, and perphenazine from 20 to 60 mg per day). The dosage of

antipsychotics then remained unchanged throughout the study. If the psychiatrists decided that a patient's response was not adequate or the patient decided to withdraw from the study, treatment was discontinued, and the last observation was carried forward to represent treatment response. Patients with adequate responses continued treatment until the end of the study.

Clinical data collection

Demographic information was collected by the self-designed report form, which included age, sex, course of schizophrenia (month), dosage of antipsychotics, height, weight at each measurement point, waist circumference, and so on. Laboratory test was conducted at baseline, the 4th week, and the 6th week, including the blood routine, blood lipids (including cholesterol, triglyceride, high-density lipoprotein, and low-density lipoprotein), and blood sugar. The severity was evaluated by the PANSS.

Genotyping

Genomic DNA was extracted with the QIAamp DNA Mini Kit (QIAGEN, Hilden, Germany). The samples were genotyped with Illumina Human Omni ZhongHua-8 Beadchips (Illumina, San Diego, CA, USA), which were designed for Chinese populations. Quality control was done before the association analysis. Samples were excluded if the genotype call rate < 98%, in the case of gender discordance, if they were first- or second-degree relatives, or if they were genetic outliers. SNPs were excluded if the minor allele frequency < 0.01, the genotype call rate < 98%, or *P*-values for Hardy-Weinberg equilibrium $< 1 \times 10^{-5}$.

Genotype imputation for the sample was done with the pre-phasing imputation stepwise approach implemented in IMPUTE (a genotype imputation method) (version 2) and segmented haplotype estimation and imputation tool (SHAPEIT) (version 2.r727). Haplotypes derived from phase I of the 1000 Genomes Project (release version 3) were used as references. SNPs with imputation quality

scores below a set threshold (info score < 0.9) were excluded from further analyses. All genomic locations are given as National Center for Biotechnology Information Build 37 coordinates.

Principal components analysis

Principal components analysis is commonly used in genetic studies to identify and correct for population structure, which can help reduce confounding effects in association studies. In this study, we used PLINK v1.90 to perform principal components analysis to summarize the genetic variation across samples. The top principal components often correlate with underlying population structure. Hence, we applied the 5 principal components as covariates (same as protocol) to correct the confounder of population structure.

Validation cohort

CATIE cohort

The CATIE schizophrenia trial was a multi-site, multi-phase randomized controlled trial with patients chronically suffering from schizophrenia. Patients were followed for up to 18 months, and data were collected from 2001 to 2004. Phase 1/1A was a randomized, controlled, double-blind comparison of 5 different treatments. The administered treatments were olanzapine, quetiapine, risperidone, and ziprasidone as second-generation antipsychotics and perphenazine as first-generation antipsychotics.

The CATIE cohort included 1460 patients with schizophrenia in the USA. After being approved by the NIMH REPOSITORY & GENOMICS RESOURCE (https://www.nimhgenetics.org/, Requested ID: 63084551a4921), we got clinical and genetic data on 766 patients with schizophrenia. Of these 741 well-genotyped patients.

Paliperidone (PAL) cohort

This cohort was a prospective, multicenter clinical trial conducted from 2021 to 2023 (the Clinical registration No. ChiCTR2100048320). Two hundred and seventy-seven patients with schizophrenia who received paliperidone for 6 weeks were recruited from 9 clinical centers, including Peking University Sixth Hospital, Beijing Anding Hospital, the Second Xiangya Hospital, Wuxi Mental Health Center, Shanghai Mental Health Center, Renmin Hospital of Wuhan University, Shandong Mental Health Center, He'nan Mental Health Center, and Xiamen Xianyue Hospital. The study complied with the Declaration of Helsinki, and the protocol was approved by the Clinical Research Ethics Committee at each center. Written informed consent was obtained before the study procedures.

Inclusion criteria. The following conditions need to be met: (1) Diagnosed with schizophrenia based on the Mini International Neuropsychiatric Interview (MINI-Plus) of the DSM-IV; (2) Aged 18 to 45 years, of any gender, of Han Chinese ancestry, and both biological parents are Han Chinese; (3) First episode with unmedicated or chronic course currently in an acute episode; (4) A total PANSS score of ≥ 60 (on a scale of $1 - 7$), requiring scores of ≥ 4 on at least 3 of the 7 positive symptoms; and (5) Obtain written informed consent from the patient or their legal guardian.

Exclusion criteria. Patients were excluded from the study if they met the following criteria: (1) Pregnant, breastfeeding, or pregnancy planning; (2) Contraindication to paliperidone; (3) With unstable physical diseases; and (4) With the following heart conditions: prolonged QTc interval (QTc ≥ 450 ms in males or QTc ≥ 470 ms in females on electrocardiogram); decompensated congestive heart failure; complete left bundle branch block.

Genotyping, imputation, and quality control. Samples were genotyped with Infinium Asian Screening Array (Illumina, San Diego, CA, USA), which was designed for East Asian populations. A total of

743,722 SNPs were successfully genotyped. Before imputation, several quality control measures were applied. Samples with call rate < 0.98, mismatched sex information, first- or second-degree relatives, and those with heterozygosity ≥ 3 standard deviations from the mean were excluded. Variants were filtered out if they had a call rate < 0.98, Hardy-Weinberg equilibrium deviations $P < 1 \times 10^{-5}$, or a minor allele frequency < 0.01. After these steps, 224 qualified samples and 289,525 variants were retained for imputation. The qualified samples and genotypes were phased using SHAPEIT (version 2). Imputation was performed using IMPUTE (version 2) with the 1000 Genomes Project East Asian reference panel. This resulted in a total of 37,662,208 variants being successfully imputed. After filtering for well-imputed variants with imputation quality scores > 0.6, 10,507,772 variants were retained. The present study additionally performed the genomic quality control for the imputed variants. Variants with a call rate < 0.05 or minor allele frequency < 0.01 were excluded, leaving a total of 3,496,532 variants for subsequent analysis.

Phenotype

Movement disorders

The Simpson-Angus Scale (SAS), the Barnes Akathisia Rating Scale (BARS), and the Abnormal Involuntary Movement Scale (AIMS) (only used the first seven items) were evaluated to assess antipsychotic-induced movement disorders. The SAS was used to assess the extrapyramidal symptom, BARS was applied to assess the akathisia, and AIMS was applied to assess the involuntary movement (the total scores of the first seven items were calculated).

Utvalg for Kliniske Undersogelser (UKU)

UKU was applied to assess the side-effects of the antipsychotics; 47 items were recorded at each visit point. The dystonia, rigidity, hypokinesia/akinesia, hyperkinesia, tremor, and akathisia were used to assess the movement disorders.

**Subgroups**	**Items**
**Psychic side effects**	Concentration difficulties
	Asthenia/lassitude/increased fatigability
	Sleepiness/sedation
	Failing memory
	Depressed mood
	Tension/inner unrest
	Increased duration of sleep
	Reduced duration of sleep
	Increased dream activity
	Emotional indifference
**Neurological side effects**	Dystonia
	Rigidity
	Hypokinesia/akinesia
	Hyperkinesia
	Tremor
	Akathesia
	Epileptic seizures
	Paraesthesias
**Autonomic side effects**	Accommodation disturbances
	Increased salivation
	Reduced salivation (dryness of mouth)
	Nausea/vomiting
	Diarrhea
	Constipation
	Micturition disturbances
	Polyuria/polydipsia
	Orthostatic dizziness
	Palpitations/Tachycardia
	Increased tendency to sweat
**Other side effects**	Rash
	Pruritus
	Photosensitivity
	Increased pigmentation
	Weight gain
	Weight loss
	Menorrhagia
	Amenorrhea

Subgroups	Items
	Galactorrhea
	Increased sexual desire
	Diminished sexual desire
	Erectile dysfunction
	Ejaculatory dysfunction
	Orgasmic dysfunction
	Dry vagina
	Headache
	Physical dependence
	Psycho dependence

Adverse effect (AE) records

AEs included 20 items as follows: akathisia, akinesia, constipation, dry mouth, gynecomastia, galactorrhea, hypersomnia, incontinence, nocturia, insomnia, menstrual irregularities, orthostatic faintness, sex drive, sexual arousal, sexual orgasm, sialorrhea, skin rash, sleepiness, urinary hesitancy, and weight gain. The akathisia and akinesia were used to assess the movement disorders.

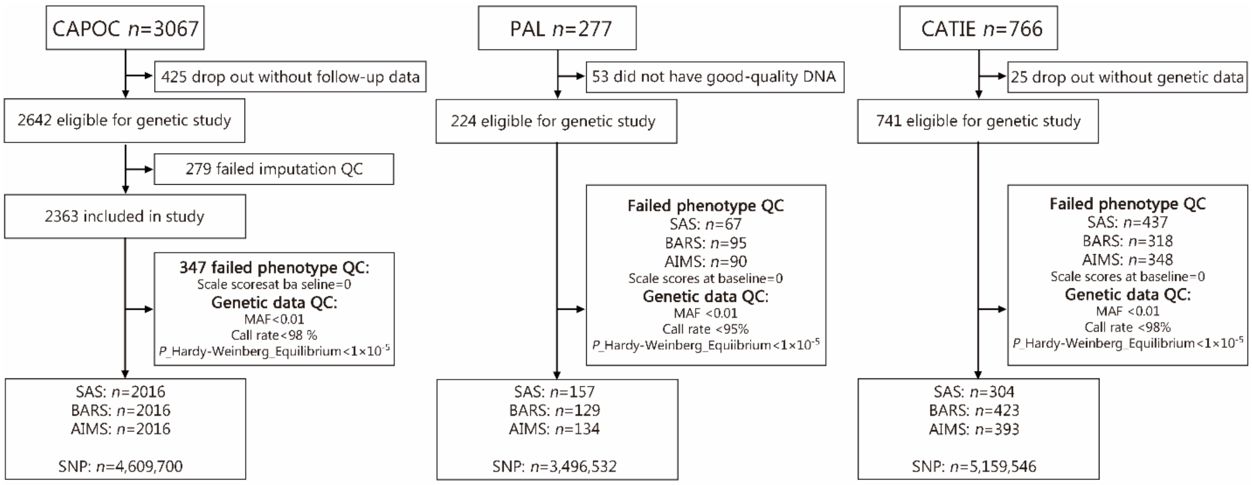

Fig. S1 Pipeline of the GWAS. CAPOC Chinese Antipsychotics Pharmacogenomics Consortium, CATIE Clinical Antipsychotic Trials of Intervention Effectiveness in Schizophrenia, PAL paliperidone, SAS Simpson-Angus Scale, BARS Barnes Akathisia Rating Scale, AIMS Abnormal Involuntary Movement Scale, GWAS genome-wide association study, QC quality control, MAF minor allele frequency, SNP single nucleotide polymorphism

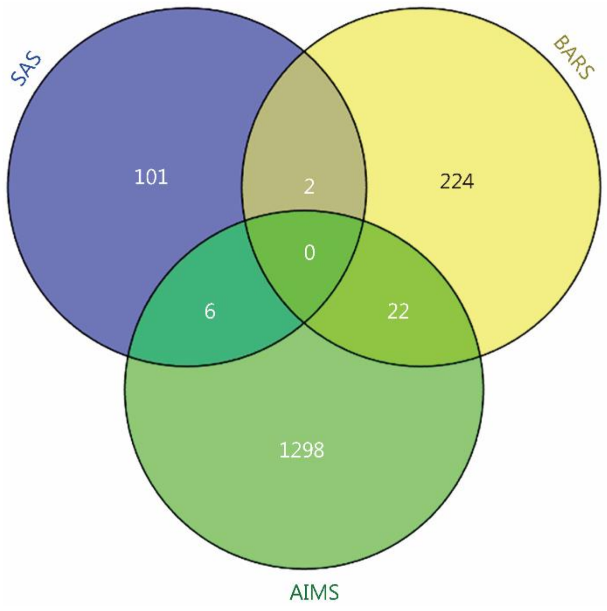

Fig. S2 Venny plot of suggestive SNPs. Overlap between SAS and AIMS: rs117082492, rs145001229 (located in *CTNND2* gene), rs74010255, rs9935338, rs6501145, rs76202841; Overlap between SAS and BARS: rs116249243, rs117097482, both SNPs located in *RAB44* gene; Overlap between BARS and AIMS: rs2244082, rs1923114, rs1329835, rs2247176, rs1329837, rs2247273, rs1126372, rs2426450, rs2247963, rs2247974, rs2248084, rs2247388, rs2145259, rs2263787, rs2247515, rs1923115, rs1923113, rs192281014, rs149643105, rs79304631, rs149124146, rs2889483. SNPs single nucleotide polymorphisms, SAS Simpson-Angus Scale, BARS Barnes Akathisia Rating Scale, AIMS Abnormal Involuntary Movement Scale

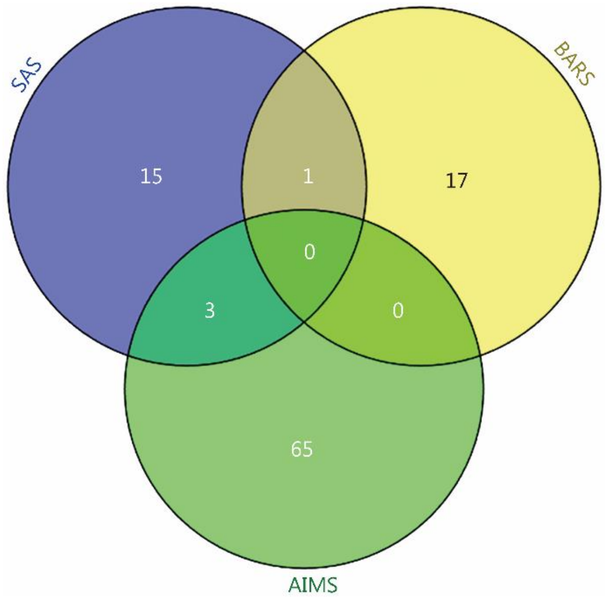

Fig. S3 Venny plot of suggestive genes. Overlap between SAS and AIMS: *CTNND2*, *STK32B*, *MYH6*; Overlap between SAS and BARS: *RAB44*. SAS Simpson-Angus Scale, BARS Barnes Akathisia Rating Scale, AIMS Abnormal Involuntary Movement Scale

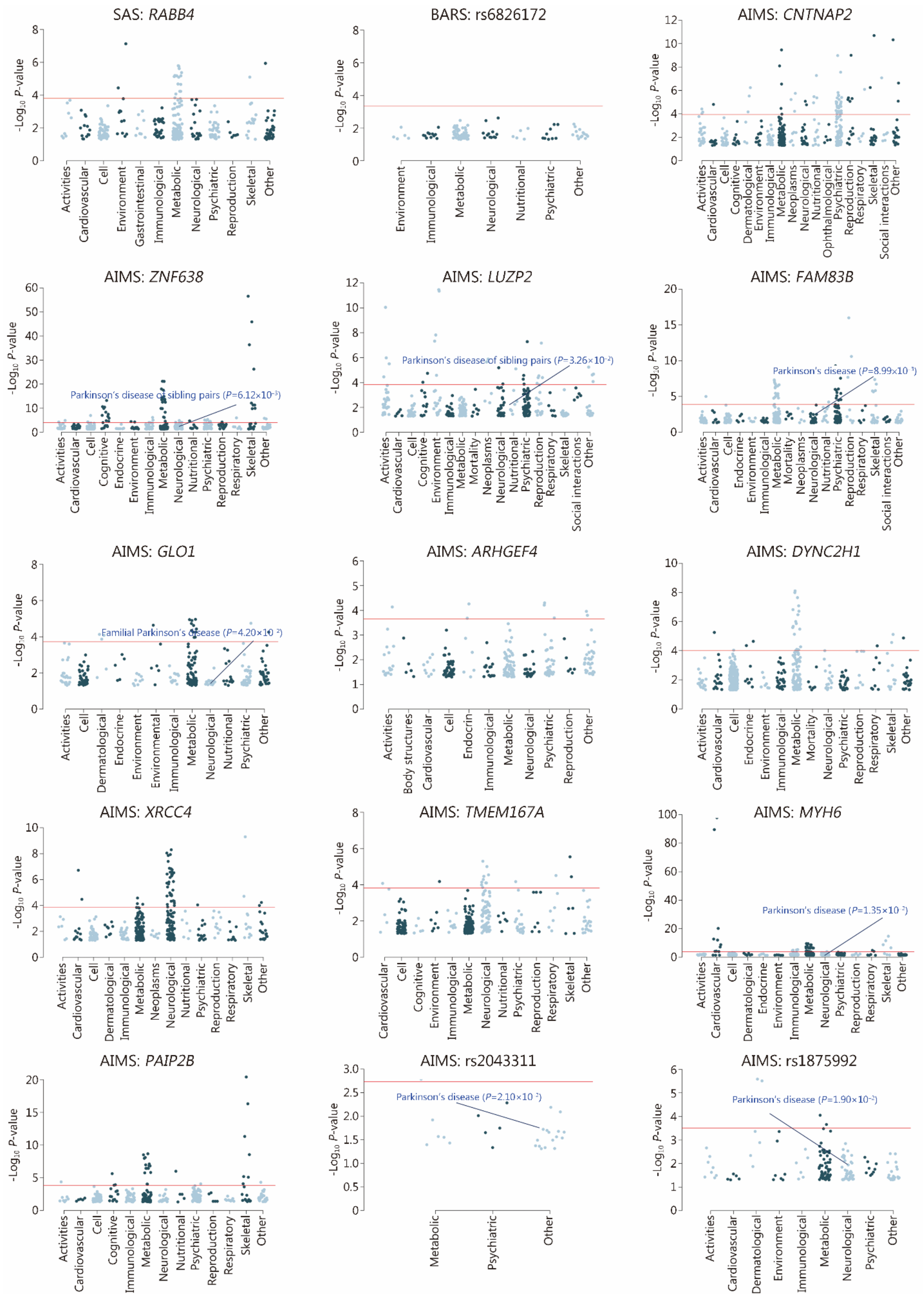

Fig. S4 PheWAS results. SAS Simpson-Angus Scale, BARS Barnes Akathisia Rating Scale, AIMS Abnormal Involuntary Movement Scale, PheWAS phenome-wide association study

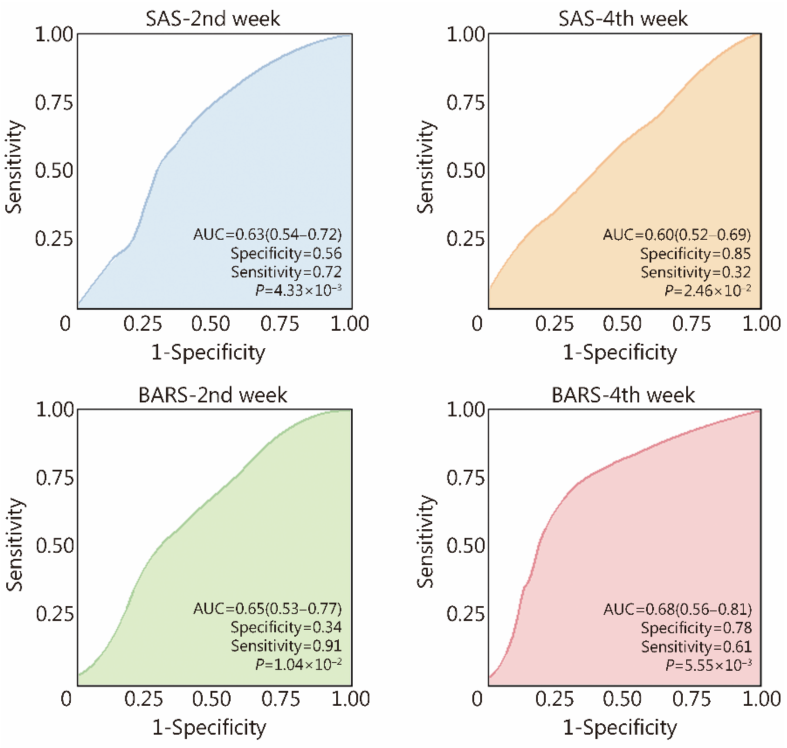

Fig. S5 Area under the curve of PRS predictors for SAS and BARS scales in the PAL cohort. PAL paliperidone, PRS polygenetic risk score, SAS Simpson-Angus Scale, BARS Barnes Akathisia Rating Scale, AUC area under the receiver operating characteristic curve
