# Supplementary material for: Substantia nigra related gene polymorphisms associated with antipsychotic-induced acute movement disorders: a genome-wide association study and multi-ancestry validation in schizophrenia
Source: Mil Med Res. 2025 Aug 19;12:50. doi: 10.1186/s40779-025-00636-w (PMC12362978; doi:10.1186/s40779-025-00636-w)
Supplement: Supplementary file 2 — Additional file 2. Protocol of the Chinese Antipsychotics Pharmacogenomics Consortium (CAPOC) cohort. [file 40779_2025_636_MOESM2_ESM.pdf]

Protocol of the Chinese Antipsychotics Pharmacogenomics Consortium (CAPOC) cohort

The genome-wide association and pharmacogenomic study of schizophrenia

Principal investigator (1)

Dai Zhang

Peking University Sixth Hospital

Hua Yuan Bei Lu Road No. 51, Beijing 100191

Principal investigator (2)

Ling-Jiang Li

The Second Xiangya Hospital, Central South University

NO. 139 Renmin Road, Changsha 41001, Hunan

Principal investigator (3)

Tao Li

West China Hospital, Sichuan University,

Chengdu 610041, Sichuan, China

Principal investigator (4)

Chuan-Yue Wang

Capital Medical University, Beijing 100088, China

Principal investigator (5)

Fu-De Yang

Beijing HuiLongGuan Hospital, Beijing 100096, China

Grant No: 2009AA022702

Chinese Clinical Trial Registry No: ChiCTR-TRC-10000934

Study summary

**Title**	The genome-wide association and pharmacogenomic study of schizophrenia
**Short Title**	Pharmacogenomic study of the effects of antipsychotics in patients with schizophrenia
**IRB No.**	2010-18
**Methodology**	Open label; Randomized.
**Study Duration**	2010 – 2012
**Study Center(s)**	Five study centers
**Objectives**	**Primary:**<br>• To investigate the relationship between the common variants and the efficacy of acute-stage antipsychotic treatment and the side effects of antipsychotics<br>**Secondary:**<br>• To compare the first-generation antipsychotics, haloperidol and perphenazine, with five commonly used atypical drugs in patients with acute-phase schizophrenia
**Number of Subjects**	3000 subjects expected to be enrolled across 5 sites
**Main Inclusion and Exclusion Criteria**	**Inclusion criteria.** (1) Chinese Han descendants; (2) Patients diagnosed with schizophrenia with first-onset or chronic disease; (3) Patients with total scores of the Positive and Negative Syndrome Scale (PANSS) score > 60; and (4) Patients who give written informed consent<br>**Exclusion criteria.** (1) Patients who are pregnant or breast-feeding; (2) Patients with contraindications to the recommended drugs; (3) Patients with severe or unstable physical diseases; (4) Patients with the following heart diseases: QT interval correction (QTc) > 450 ms in males, or QTc > 470 ms in females; (5) Decompensated and congestive heart failure; and (6) Complete left bundle branch block delay
**Investigational product (drug, biologic, device, etc.)**<br>**For the device include the planned use**<br>**For drug, food, cosmetic, etc., include the dose, route of administration, and dose regimen**	Olanzapine doses could range from 5 to 20 mg per day, risperidone from 2 to 6 mg per day, quetiapine from 400 to 750 mg per day, aripiprazole from 10 to 30 mg per day, ziprasidone from 80 to 160 mg per day, haloperidol from 6 to 20 mg per day, and perphenazine from 20 to 60 mg per day
**Statistical methodology**	We evaluate the associations between allele dosages and treatment response using linear regression under an additive genetic model implemented in PLINK v1.07. Gender, age, site of collection, and the first 5 principal components of population structure and other factors are used as covariates

**Safety evaluations**	Site investigators will be responsible for monitoring the safety of study participants. They must alert the Medical Officer of any event that seems unusual. The investigators will be responsible for the appropriate medical care of study participants during the study, in connection with study procedures
**Data and safety monitoring plan**	Principal investigators (PIs) will be responsible for monitoring the data quality and the ongoing safety of subjects

Background and study rationale

This study will be conducted in full accordance with all applicable Peking University Research Policies and Sichuan University Procedures and all applicable Chinese laws and regulations.

Introduction

Antipsychotics are the cornerstone for clinical treatment of schizophrenia, but patients show variations in response to antipsychotic drug treatment. Previous studies demonstrated that genetic components play a vital role in individual differences and identified several candidate genes for the variability of treatment response. However, many common variants were identified in Caucasian samples. Additional risk variants may be detected when increasing the sample size in different samples. Therefore, the CAPOC was set to understand how genetic variants influence antipsychotic treatment response and side effects.

Background and relevant studies

Schizophrenia is a chronic and severe mental disease that affects approximately 1% of the world's population. Currently, antipsychotic drugs are the mainstay of schizophrenia and reduce the risks of clinical deterioration and psychotic relapse. Three-quarters show low compliance for antipsychotic medications because of ineffectiveness or side effects [1]. In turn, this may cause clinical exacerbation or psychotic relapse, which often results in hospitalization and causes a considerable burden on patients and their families. Antipsychotics correspond to a considerable fraction of healthcare costs in most developed countries. A modest improvement in outcomes may provide great benefits for society. Therefore, it is important to understand the biological mechanisms of antipsychotic treatment response.

The heritability for antipsychotic drug responses is extremely limited because of difficulties recruiting twin pairs who have received the same antipsychotic treatment. Several twin and family studies suggest that the response to antipsychotic treatment is a heritable trait. The studies on monozygotic twins observed a similar response to treatment with antipsychotics and similar levels of

antipsychotic-induced weight gain [2]. Given that schizophrenia has a high heritability, it is likely that there is a substantial genetic component to individual differences in treatment response.

In the past decades, pharmacogenetics research has succeeded in identifying genetic variants associated with variability in antipsychotic treatment. These studies have focused on encoding drug targets (pharmacodynamic candidates) or on involvement in the metabolism of the drug itself (pharmacokinetic candidates). Pharmacodynamic candidates have shown positive associations with treatment response, such as dopamine receptors D_2 (*DRD2*) and D_3 (*DRD3*) and serotonin receptor genes, including 5-hydroxytryptamine receptor 2A (*HTR2A*) and 2C (*HTR2C*). Pharmacokinetic candidates include the cytochrome *P450* genes and *ABCB1* transporter genes. In contrast to candidate gene-based methods, Genome-wide association studies (GWASs) could identify candidate variants without introducing prior hypothesis bias. Several groups have performed GWASs to identify candidate biomarkers for treatment response and side effects of antipsychotics. Currently, the researchers mainly use the genotyping data from the original Clinical Antipsychotic Trials of Intervention Effectiveness (CATIE) study and focus on various phenotypes. For example, McClay et al. [3] used PANSS subscales as well as PANSS total as measures of treatment response and identified 2 intragenic.

Single-nucleotide polymorphisms (SNPs) were close to the significant GWAS-based threshold, including rs7968606 (*ANKS1B*) for negative symptom improvement with olanzapine and rs17727261 (*CNTNAP5*) for negative symptom improvement with risperidone. Furthermore, they focused on neurocognition and used 5 neurocognitive domains and a composite neurocognitive score as drug response indicators. They found that *DRD2* mediated the effects of olanzapine on working memory, *LPHN3* and *CLDN1* mediated the effects of quetiapine on working memory. Clark et al. [4] used clinical global impression of response scales as a measure of treatment response. They found that *PDE4D* mediated the effects of quetiapine on patient-reported severity, *TJP1* mediated the effects of risperidone on patient-reported severity, and *PPA2* mediated the effects of risperidone on clinician-reported severity.

Numerous studies have shown that antipsychotic treatment is often associated with medical complications [5]. The first-generation antipsychotics (FGAs) could relieve a substantial proportion of schizophrenia patients to improve or relapse frequently; however, they are often accompanied by significant side effects, including extrapyramidal symptoms and tardive dyskinesia. The second-generation antipsychotics (SGAs) show lower affinity for the DRD2 and relatively greater affinities for other neuroreceptors, but they are associated with a variety of metabolic side effects such as dyslipidemia, elevated glucose levels, and weight gain. Pharmacogenetic research efforts have focused on the identification of genetic variants contributing to individual variability regarding several antipsychotic-related phenotypes. Previous studies have reported using the CATIE sample to perform GWAS for antipsychotic induced side effects. Adkins et al. [6] performed a GWAS in the CATIE sample for several metabolic side effects. *GPR98* and *NR3C1* genes were respectively found to be associated with hemoglobin A1c (HbA1c) levels in schizophrenia patients treated with olanzapine and risperidone. The *MEIS2* gene was identified to be correlated with waist and hip circumferences for patients treated with risperidone. Aberg et al. [7] performed a GWAS for extrapyramidal side effects in the CATIE sample. They found 3 SNPs, rs17022444, rs7669317, and rs2126709 (*ZNF202*) were significantly associated with movement-related adverse antipsychotic effects.

However, most common variants associated with treatment response and side effects of antipsychotics were identified in large samples of Caucasian ancestry. The associated variants identified in populations of Caucasian ancestry might not be totally significant in other ancestry groups because of underlying genetic heterogeneity. Therefore, large-scale studies in Chinese and other non-Caucasian populations are needed not only to evaluate whether the previously reported genetic variants could be generalized to the non-Caucasian population, but also to identify new associated variants for antipsychotics.

In recent years, the role of rare variants has been recognized in several neuropsychiatric disorders, including schizophrenia, intellectual disability, autism spectrum disorder, and serotonin reuptake inhibitor treatment response in major depression. Since most drugs exert their effects through protein

binding, whole-exome sequencing offers a cost-effective strategy for investigating rare functional variants in drug response studies. In addition, rare variants in specific genes or gene sets may define a disease subtype that is relatively non-responsive to the standard drug treatment for a disorder.

Overview of trial design

This study is a randomized controlled trial of up to 3000 patients with schizophrenia involving the following medications: aripiprazole, olanzapine, quetiapine, risperidone, ziprasidone, haloperidol, or perphenazine. Patients will be followed for up to 6 weeks. Patients are enrolled from members from 5 research centers (Peking University Sixth Hospital, West China Hospital of Sichuan University, the Second Xiangya Hospital of Central South University, Beijing Anding Hospital Affiliated to Capital Medical University, and Beijing Huilongguan Hospital). The Consortium, which leads 32 psychiatric hospitals in China, aims to understand the relationship between genetic variants and antipsychotic treatment responses in patients with schizophrenia.

Study objectives

Primary objective

To investigate the relationship between the common variants and the efficacy of acute-stage antipsychotic treatment or the side effects of antipsychotics.

Secondary objectives

To compare the first-generation antipsychotics, haloperidol and perphenazine, with 5 commonly used atypical drugs in patients with acute-phase schizophrenia.

Investigational plan

General design

Patients will be enrolled from 5 research centers (the Sixth Hospital of Peking University, the Second Xiangya Hospital of Central South University, West China Hospital of Sichuan University, Beijing Anding Hospital, and Beijing Huilongguan Hospital), comprising 32 hospitals across China. As is

known, schizophrenia is mainly diagnosed based on subjective symptoms. Therefore, considering the importance of coherence, we will conduct 5 trainings for the psychiatrists in 32 hospitals respectively. The content of training included research protocols, diagnostic criteria, and instruments, scales for assessment of symptoms and side effects, blood sample collection, and evaluation of inter-rater reliability as well.

Patients were initially randomly assigned to 7 groups, including aripiprazole, olanzapine, quetiapine, risperidone, ziprasidone, haloperidol, and perphenazine groups. Randomization was performed as complete randomization with a 2:2:2:2:2:2:1:1 allocation. Group assignment was determined by a Microsoft Excel randomization generator. The random allocation sequence was generated by a trained research assistant. Then, we performed several assessments of the baseline at the start of the study. The patients who meet the criterion are followed for up to 6 weeks or until treatment is discontinued for any reason.

Screening phase. Subjects will be recruited through the oral explanation of the study. Interested subjects will be consented to verbally over the interview by the clinical doctor. A series of questions will be asked during the interview to determine if the potential subject is within the correct age range and geographic location. Potential subjects eligible based on these criteria will be brought in for a second screening visit, at which time labs will be conducted as described in the inclusion/exclusion criteria section of the protocol. Written consent will be obtained before the screening labs are conducted.

Allocation to the interventional group. The randomization algorithm will be built into the electronic data capture system. Once the randomization form is entered into the system and saved, the back-end algorithm will run, and a participant will be assigned a kit number corresponding to either drug. The master list of kit number assignments will be kept by the data coordinating center in a password-protected and encrypted laptop.

Study endpoints

Primary study endpoints. The primary endpoint will be the % change in PANSS score between the

baseline visit and the last visit. The % change in PANSS score is defined as follows, PANSS reductive ratio (%) = (PANSS baseline score – PANSS follow-up score)/(PANSS baseline score – 30) × 100%.

Secondary study endpoints. We evaluate 2 kinds of phenotypes for side effects of antipsychotics: dichotomous phenotype and continuous phenotype. (1) Dichotomous phenotype: patients with metabolic syndromes (MetS) vs. patients without MetS. Diagnosis of MetS is based on the definition by the International Diabetes Federation Chinese criteria. Those with central obesity assessed by waist circumference ≥ 90 cm in males and ≥ 80 cm in females, plus 2 of the following: elevated triglycerides level ≥ 1.7 mmol/L (or use of a fibrate), high-density lipoprotein < 1.03 mmol/L in males and < 1.29 mmol/L in females (or use of a statin), fasting glucose ≥ 5.6 mmol/L (or use of an antidiabetic drug) and systolic arterial blood pressure ≥ 130 mmHg and/or diastolic arterial blood pressure ≥ 85 mmHg (or use of an antihypertensive drug) were identified as having MetS. (2) Continuous phenotype: quantifying antipsychotic-induced change in the assessments as described above, for example, change in body mass index, blood lipids, glucose, and hemoglobin.

Study population and duration of participation

Inclusion criteria

Individuals included for the present study had a diagnosis of schizophrenia determined by the Structured Clinical Interview of Diagnostic and Statistical Manual of Mental Disorders, fourth edition (DSM-IV), were 18 − 45 years old, and of Han Chinese ancestry, had a total score of the PANSS > 60, and at least 3 positive items cored > 4, were physically healthy and had all laboratory parameters within normal limits, had a condition appropriate for treatment with an oral medication, and provided informed consent. First-episode or relapsed patients with schizophrenia were enrolled from the inpatient departments of the psychiatric hospitals of the CAPOC project. The patients were without prior antipsychotics for more than 1 week before enrollment. Within 2 weeks before the enrollment and throughout the study, patients should not have taken drugs inducing or inhibiting liver enzymes. After the study entry, the antipsychotics remained unchanged throughout the study. All participants are asked to appoint a family member or close friend who is involved with the informed consent

discussion and assists the patient with decision making.

Exclusion criteria

Patients were excluded from the study if they met the following criteria. (1) Diagnosed as schizoaffective disorder, schizoaffective disorder, delusional disorder, brief psychotic disorder, schizophreniform disorder, psychosis associated with substance use or medical conditions, mental retardation, pervasive developmental disorder, delirium, dementia, amnesia, or other cognitive disorders. (2) Patients with severe unstable physical diseases, such as diabetes, thyroid diseases, hypertension, and cardiac diseases. (3) Patients with malignant syndrome and acute dystonia. (4) Patients with well-documented histories of epilepsy and hyperpyretic convulsions. (5) Patients with a DSM-IV diagnosis of alcohol and drug dependence. (6) Patients who require long-acting injectable medication to maintain treatment adherence. (7) Patients regularly treated with clozapine for treatment over the past month. (8) Patients who were treated with electroconvulsive therapy over the last month. (9) Patients with a history of drug-induced malignant syndrome. (10) Patients who have serious suicide attempt, or severe excitement and agitation situations. (11) Patients with the following abnormality of liver or renal function examination test: aspartate transaminase ≥ 80 U/L; alanine transaminase ≥ 80 U/L; blood urea nitrogen ≥ 9.75 mmol/L; urine creatinine ≥ 21.6 mmol/d. (12) Patients who have no legal guardian. (13) Patients with the following cardiac conditions: QTc prolongation (screening electrocardiogram with QTc > 450 ms for men, QTc > 470 ms for women); history of congenital QTc prolongation; recent myocardial infarction (< 6 months). (14) Women who are pregnant or breastfeeding. (15) Patients with a contraindication to any of the drugs to which they might be assigned.

Subject recruitment

The members of CAPOC from 5 research centers (the Sixth Hospital of Peking University, the Second Xiangya Hospital of Central South University, West China Hospital of Sichuan University, Beijing Anding Hospital, and Beijing Huilongguan Hospital) are responsible for patient recruitment. Patients with schizophrenia are recruited through inpatient units from 5 clinical research centers. All

participants are asked to appoint a family member who is involved with the informed consent discussion and assists the patient with decision making. All recruitment materials, which will be seen by potential participants, need to be approved by the institutional review board of each center.

Duration of study participation

The duration of the study subjects' participation, including screening, study intervention phase, and any follow-up period, is about 6 weeks.

Total number of subjects and sites

Five hundred subjects will be enrolled from each of the 5 centers.

Vulnerable populations

Pregnant women, fetuses, neonates, or prisoners are not included in this research study.

Study intervention (study drug, device, biologic, vaccine, food, etc.)

Description

Patients will take a tablet, including aripiprazole, olanzapine, quetiapine, risperidone, ziprasidone, haloperidol, or perphenazine).

Intervention regimen

Patients who met inclusion criteria were randomly assigned to 6 groups (aripiprazole, olanzapine, quetiapine, risperidone, ziprasidone, and one of the first-generation antipsychotics, haloperidol or perphenazine). Within the first 2 weeks after enrollment, the psychiatrists from the CAPOC study adjusted drug dosages based on the treatment effectiveness following the study protocol (olanzapine 5 − 20 mg/d, risperidone 2 − 6 mg/d, quetiapine 400 − 750 mg/d, aripiprazole 10 − 30 mg/d, ziprasidone 80 − 160 mg/d, haloperidol 6 − 20 mg/d, and perphenazine 20 − 60 mg/d). The dosage of antipsychotics then remained unchanged throughout the study. The patients were evaluated by a participating psychiatrist at baseline, and weeks 2, 4, and 6, and their PANSS scores were recorded.

Receipt

The investigational drug will be obtained from the CAPOC hospital.

Storage

The investigational drug will be stored in the pharmacy of each research center at room temperature, keeping it in a cool, dry room.

Blinding

The random allocation sequence was generated by a trained research assistant who had no further role in the trial and was concealed until after baseline assessments. The researchers doing both the baseline and the follow-up assessments were masked to the group assignments of each participant. Patients and psychiatrists were unmasked to the assigned antipsychotics.

Study procedures

Below is a schedule of events for the CAPOC project.

Item	First interview (Baseline)	Second interview (14th day)	Third interview (28th day)	Fourth interview (42nd day)
Informed consent	*			
Subjects screen	*			
General information	*			
Symptoms and medical history	*			
DSM-IV-TR (SCID)	*			
Body and nervous system examination	*	*	*	*
Vital signs	*	*	*	*
Weight and waist circumference	*	*	*	*
PANSS and CGI	*	*	*	*
SAESS, BARS, AIMS, and UKU	*	*	*	*
Laboratory examination	*		*	*
Electrocardiograph (ECG)	*		*	*
Blood drawing for genotyping	*			
Plasma concentration			*	*
Concomitant medications monitoring	*	*	*	*
Adverse events (AEs)	*	*	*	*
Form for the ending				*

PANSS Positive and Negative Syndrome Scale, *CGI* clinical global impression, *SAESS* Simpson-Angus Extrapyramidal Signs Scale, *BARS* Barnes Akathisia Rating Scale, *AIMS* Abnormal Involuntary Movement Scale,

UKU Udvalg for Kliniske Under-sogelser, *SCID* Structured Clinical Interview for Diagnostic and Statistical Manual of Mental Disorders, *DSM-IV-TR* Diagnostic and Statistical Manual of Mental Disorders, Fourth Edition, Text Revision

Screening

Participants who meet the inclusion criteria, which are broad and nonrestrictive, will be recruited from clinical facilities that are representative of the sites where persons with schizophrenia receive mental health care in China. The sample will include 3000 patients representing the schizophrenias. The screening visit will consist of screening tests, patient history, laboratory tests, and physical and psychiatric examinations. The baseline visit will occur within 21 d of the screening visit. The results of all screening tests will be reviewed before the baseline visit. All inclusion and exclusion criteria will be verified at the baseline visit for patients to be randomly assigned to a treatment group. Patients who do not meet all enrollment criteria at the baseline visit will not be randomized, and their participation in the study will end. Patients who meet all criteria will be enrolled in the study and will be randomized to a study medication.

Study intervention phase

The treatment phase begins a 6-week treatment period. Qualified patients will be assigned at the baseline visit to one of the treatment groups according to their clinical situation.

- 3000 patients will be randomly assigned to 1 of 7 treatment conditions for up to 6 weeks;
- 500 patients will begin open-label treatment with perphenazine or haloperidol; 500 patients will begin open-label treatment with olanzapine;
- 500 patients will begin open-label treatment with quetiapine; 500 patients will begin open-label treatment with risperidone; 500 patients will begin open-label treatment with aripiprazole; 500 patients will begin open-label treatment with ziprasidone.

Baseline visit

A physical exam must be conducted for screening purposes, but if 1 was conducted within the past 30 d for the standard of care purposes, this can be used for screening/eligibility.

- Informed consent
- Subjects screen
- Randomization
- General information
- Symptoms and medical history
- Vital signs
- Weight and waist circumference
- Laboratory tests
- PANSS and CGI
- Adverse reaction assessment
- Concomitant medications monitoring
- ECG
- Blood drawing for genotyping

Visit 2

List of all the procedures that will take place at study visit 2

- Vital signs
- Weight and waist circumference
- PANSS and CGI
- Adverse reaction assessment
- Concomitant medications monitoring

Visit 3

- Vital signs
- Weight and waist circumference
- Laboratory tests
- PANSS and CGI
- Adverse reaction assessment

- Concomitant medications monitoring
- ECG

Visit 4

- Vital signs
- Weight and waist circumference
- Laboratory tests
- PANSS and CGI
- Adverse reaction assessment
- Concomitant medications monitoring
- ECG

Subject withdrawal

Subjects may withdraw from the study at any time without impact on their care. They may also be discontinued from the study at the discretion of the investigator for lack of adherence to intervention or study procedures or visit schedules, AEs, or other reasons. The investigator may also withdraw subjects who violate the study plan, or to protect the subject for reasons of safety, or administrative reasons. It will be documented whether each subject completes the clinical study. If treatment was discontinued, the last observation was carried forward to represent treatment response.

Study evaluations and measurements

Medical record review

A list of the medical record review will be abstracted from the medical chart. Patients were excluded from the study if they were diagnosed with schizoaffective disorder, delusional disorder, brief psychotic disorder, schizophreniform disorder, psychosis associated with substance use or medical conditions, learning disability, pervasive developmental disorder, delirium, dementia, amnesia, or other cognitive disorders; had severe, unstable physical diseases (such as diabetes, thyroid diseases, hypertension, and cardiac diseases), malignant syndrome or acute dystonia, well documented

histories of epilepsy and hyperpyretic convulsion, a DSM-IV diagnosis of alcohol or drug dependence, or a history of drug-induced neuroleptic.

Physical examinations

The baseline evaluations include the medical history, physical examination, demographic characteristics (age, gender, and race), and other information that will be collected.

Laboratory evaluations

Fasting blood glucose level, hemoglobin level, lipid profile (total cholesterol, high-density lipoprotein cholesterol, triglycerides) level, complete blood count, and serum prolactin level.

Pregnancy testing

A urine pregnancy test will be performed for female subjects.

Statistical plan

Sample size and power determination

We calculate the power to detect the observed association findings under an additive genetic model using Quanto (version 1.2.4).

Statistical methods

Using PLINK v1.07, we evaluated the association between allele dosages and the dichotomous phenotype by logistic regression, and the association between allele dosages and the quantitative phenotype by linear regression. We also used gender, age, site of collection, and the first 5 principal components of population structure as covariates. We respectively examine the associations between the common variants and side-effects in the pooled antipsychotics treatment groups (7 drugs) and single antipsychotics.

For rare variants, variant-based, gene-based, and gene set-based association analyses will be used and statistical tools, including PLINK/SEQ, KGGSEQ, and RVTEST, etc., in addition, the data of Kyoto Encyclopedia of Genes and Genomes (KEGG) and Mouse Genome Informatics (MGI) will

be used to explore gene set analyses.

Baseline data. Baseline and demographic characteristics will be summarized by standard descriptive statistics (including mean and standard deviation for continuous variables such as age and standard percentages for categorical variables such as gender).

Efficacy analysis. We use the PANSS reductive ratio for the evaluation of acute-stage treatment response to antipsychotic medications. The reductive ratio is defined as follows: PANSS reductive ratio (%) = (PANSS baseline score – PANSS follow-up score)/(PANSS baseline score – 30) × 100%.

Interim analysis. An interim analysis will be performed after the first 300 subjects are enrolled in the trial. At this time, the safety and tolerability of the study dose will be assessed, and if deemed safe and appropriate, enrollment will continue.

Safety analysis. All subjects who entered the study and were randomized at the baseline visit will have detailed information collected on AEs for the overall study safety analysis.

Safety and AEs

Definitions

AE. AE is any symptom, sign, illness, or experience that develops or worsens in severity during the study. Intercurrent illnesses or injuries should be regarded as AEs. Abnormal results of diagnostic procedures are AEs if the abnormality:

- results in study withdrawal
- is associated with a serious AE
- is associated with clinical signs or symptoms
- leads to additional treatment or to further diagnostic tests
- is considered by the investigator to be of clinical significance

Serious AE. AEs are classified as serious or non-serious. A serious AE is any AE that is:

- fatal

- life-threatening
- requires or prolongs hospital stay
- results in persistent or significant disability or incapacity
- a congenital anomaly or birth defect
- an important medical event. Important medical events are those that may not be immediately life-threatening, but are clearly of major clinical significance.
- they may jeopardize the subject and may require intervention to prevent one of the other serious outcomes noted above. For example, drug overdose or abuse, a seizure that did not result in inpatient hospitalization, or intensive treatment of bronchospasm in an emergency department would typically be considered serious.
- all AEs that do not meet any of the criteria for serious should be regarded as non-serious AEs.

AE process and handling

At each contact with the subject, the investigator will seek information on AEs by specific questioning and, as appropriate, by examination. Information on all AEs will be recorded immediately in the source document and in the appropriate AE module of the case report form. All related signs, symptoms, and abnormal diagnostic procedures results should be recorded in the source document, though they should be grouped under 1 diagnosis.

All AEs occurring during the study period will be recorded. The clinical course of each event will be followed until resolution, stabilization, or until it has been determined that the study intervention or participation is not the cause. Serious AEs that are still ongoing at the end of the study period will be followed up to determine the outcome. Any serious AE that occurs after the study period and is possibly related to the study intervention or study participation will be recorded and reported immediately.

Differences between protocol and trial

We stated the difference made after trial commencement here.

Due to the severity of symptoms in the enrolled patients and the urgent need for treatment during

the acute phase, no restriction on the use of antipsychotic medications before enrollment was made. The screening visit and baseline visit were completed in a single session. Consequently, after subjects were recruited through an oral explanation of the study, those meeting the eligibility criteria were scheduled for a visit. During this visit, screening tests, patient history, laboratory tests, and physical and psychiatric examinations were conducted. Eligible participants were enrolled and randomized to study medication.

References

1. Samara MT, Nikolakopoulou A, Salanti G, Leucht S. How many patients with schizophrenia do not respond to antipsychotic drugs in the short term? An analysis based on individual patient data from randomized controlled trials. Schizophr Bull. 2019;45(3):639-46.
2. Zhang JP, Malhotra AK. Pharmacogenetics and antipsychotics: therapeutic efficacy and side effects prediction. Expert Opin Drug Metab Toxicol. 2011;7(1):9-37.
3. McClay JL, Adkins DE, Aberg K, Stroup S, Perkins DO, Vladimirov VI, et al. Genome-wide pharmacogenomic analysis of response to treatment with antipsychotics. Mol Psychiatry. 2011;16(1):76-85.
4. Clark SL, Souza RP, Adkins DE, Aberg K, Bukszár J, McClay JL, et al. Genome-wide association study of patient-rated and clinician-rated global impression of severity during antipsychotic treatment. Pharmacogenet Genomics. 2013;23(2):69-77.
5. Lally J, MacCabe JH. Antipsychotic medication in schizophrenia: a review. Br Med Bull. 2015;114(1):169-79.
6. Adkins DE, Aberg K, McClay JL, Bukszár J, Zhao Z, Jia P, et al. Genomewide pharmacogenomic study of metabolic side effects to antipsychotic drugs. Mol Psychiatry. 2011;16(3):321-32.
7. Aberg K, Adkins DE, Bukszár J, Webb BT, Caroff SN, Miller DD, et al. Genomewide association study of movement-related adverse antipsychotic effects. Biol Psychiatry. 2010;67(3):279-82.
